# Current status (as of end of 2020) of marine alien species in Turkey

**DOI:** 10.1371/journal.pone.0251086

**Published:** 2021-05-04

**Authors:** Melih Ertan Çinar, Murat Bilecenoğlu, M. Baki Yokeş, Bilal Öztürk, Ergün Taşkin, Kerem Bakir, Alper Doğan, Şermin Açik

**Affiliations:** 1 Department of Hydrobiology, Faculty of Fisheries, Ege University, Bornova, İzmir, Turkey; 2 Department of Biology, Faculty of Arts & Sciences, Aydın Adnan Menderes University, Aydın, Turkey; 3 AMBRD Doğa Bilimleri, Şişli, İstanbul, Turkey; 4 Department of Biology, Faculty of Arts & Sciences, Celal Bayar University, Manisa, Turkey; 5 Institute of Marine Sciences and Technology, Dokuz Eylül University, İnciraltı, İzmir, Turkey; Universita degli Studi di Genova, ITALY

## Abstract

The 2020’s update of marine alien species list from Turkey yielded a total of 539 species belonging to 18 taxonomic groups, 404 of which have become established in the region and 135 species are casual. A total of 185 new alien species have been added to the list since the previous update of 2011. The present compilation includes reports of an ascidian species (*Rhodosoma turcicum*) new to the marine fauna of Turkey and range extensions of six species. Among the established species, 105 species have invasive characters at least in one zoogeographic region, comprising 19% of all alien species. Mollusca ranked first in terms of the number of species (123 species), followed by Foraminifera (91 species), Pisces (80 species) and Arthropoda (79 species). The number of alien species found in seas surrounding Turkey ranged from 28 (Black Sea) to 413 (Levantine Sea). The vectoral importance of the Suez Canal diminishes when moving from south to north, accounting for 72% of species introductions in the Levantine Sea *vs*. only 11% of species introductions in the Black Sea. Most alien species on the coasts of Turkey were originated from the Red Sea (58%), due to the proximity of the country to the Suez Canal. Shipping activities transported 39% of alien species, mainly from the Indo-Pacific area (20%) and the Atlantic Ocean (10%). Misidentified species (such as *Pterois volitans*, *Trachurus declivis*, etc.) and species those classified as questionable or cryptogenic were omitted from the list based on new data gathered in the last decade and expert judgements. The documented impacts of invasive species on socio-economy, biodiversity and human health in the last decade as well as the legislation and management backgrounds against alien species in Turkey are presented.

## Introduction

The human mediated translocations of marine species have drastically altered the biodiversity and food web structures of the Mediterranean Sea at an unprecedented rate. This semi-enclosed ecosystem is not only a biodiversity hotspot owing to the relatively high levels of endemism and endangered taxa [1], but also one of the prominent hotspots of marine bioinvasions on earth [2, 3]. Monitoring the spread of alien organisms is thus among the most immediate nature conservation issues to be faced.

The recent alien diversity estimates, excluding questionable and cryptogenic species, indicate the presence of nearly 900 species, 75% of which have established successfully breeding populations in the region [4, 5]. Although there were serious concerns that the recent expansion of the Suez Canal by 2015 could trigger a huge wave of invasions [6], the annual rate of introductions in the Mediterranean seems to be in a decreasing trend, currently with no rational explanations, except for the survey intensity over years [7]. One new species record in every two weeks was given for the period of 2011–2012 [8], which sharply has decreased to a level of 4 sp./year between 2017 and 2019, regardless of the pathway [5]. Alien species diversity displays significant differences among basins of the Mediterranean Sea. Highest number of species were recorded from the eastern basin, dominated by Indo-Pacific taxa introduced via the Suez Canal, while almost 6 folds lower diversity figures exist at the western part, in which fouling and ballast water transportation along shipping lines appear to be the main vector [8, 9].

Located at the junction of three continents, Turkey is among the most impacted countries from bioinvasions for a couple of reasons. The distance between Suez Canal and Iskenderun Bay is almost 350 nautical miles and such proximity facilitates alien species that traversed the canal to easily flux through the prevailing counter-clockwise Mediterranean currents. Moreover, there is a dense maritime traffic in the Turkish Straits System (Çanakkale and İstanbul Straits); number of vessels passing through the İstanbul Strait was over 40,000 vessels during 2019 [10], which is significantly higher from the Suez Canal where some 17,000 vessels pass annually [6]. Although responsible from a minor magnitude of impact, existence of several coastal aquaculture facilities is also a known potential vector for species introductions.

So far, the alien biota of Turkey has been compiled by a group of national experts in two comprehensive checklists [7, 11]. Obviously, both lists are currently out of date, since i) several new records were given during the last decade (*Berthellina citrina*, *Styela clava*, *Equulites popei*, etc.) [12–14], ii) the establishment success of some species have changed (for example *Parupeneus forsskali* remained as a casual species for over a decade after its first record [15], which currently invaded the entire northern Levant shores), iii) the distribution ranges of already recorded species expanded (the veteran alien fish *Stephanolepis diaspros* has penetrated to the Sea of Marmara [16]), iv) taxonomical revisions reveal misidentifications (*Lagocephalus guentheri* was erroneously misidentified as *L*. *spadiceus* [17]), v) native/alien status of some species changed (as in the case of *Oculina patagonica*, which was previously considered as an alien species, now shifted to native category [18]), vi) some Red Sea/Indo-Pacific species have recently been described as new species from the Mediterranean Sea (*Hazeus ingressus* [19] and *Chrysaora pseudoocellata* [20]).

The Convention on Biological Diversity calls on in its Article 8 (h) each Contracting Party, as far as possible and as appropriate “*to prevent the introduction of*, *control or eradicate those alien species which threaten ecosystems*, *habitats or species*”, in which dealing with the ongoing threat through available measures relies primarily on updated inventories [21]. Avoiding errors and inaccuracies in species listing processes are also vital for many areas in conservation biology [22] and regional datasets of marine alien species are of utmost importance for the success of regulation on the prevention and management of invasive taxa [23]. It is therefore necessary to review, validate and update the list of alien species in accordance with current scientific information. We here present a comprehensive treatise on the recent status of all alien marine taxa reported from Turkey, a core area drastically impacted by bioinvasions. Reducing geographical data gaps is a prerequisite for mitigation actions that will also serve as a baseline for the development of open data infrastructures, presently lacking in Turkey.

## Material and methods

The present study is an update of the alien species list given by [7], based on new information on the taxonomic entities and distributions of alien species. The present study also adds new information about the presence of alien species along the coasts of Turkey. As having different oceanographic characteristics, the current status of alien species in the seas (Black Sea, Sea of Marmara, Aegean Sea and Levantine Sea) surrounding Turkey were evaluated separately. The first collection year of the species were extracted in the respective papers, but if there is no information about it, the year of sample collection was determined by making a personal interview with the corresponding author. For example, as there is no indication on the collection date of the hydroid *Eudendrium merulum* [24], the publication date (2000) of the paper was regarded as the collection date of the species in the Sea of Marmara and Aegean Sea [25]. However, after interviewed with the corresponding author (A.C. Marques), the collection date of the species was corrected as 1953 for the Sea of Marmara and 1977 for the Aegean Sea.

Some species previously regarded as alien species in the region were excluded from the list because of several reasons including misidentification, or misinterpretation of its origin and pathway of arrival. The species excluded from the alien species list given by [7] and the reasons for their eliminations are presented.

Alien species were grouped into two categories, namely established and casual alien species [26]. Species that formed self-maintaining populations with at least two records in the area (three records for fish) spread over time and space are classified as established species, while those having been recorded only once (no more than twice for fish) with no evidence of self-sustaining populations are classified as casual species. If detected in countries (Syria, Lebanon, Israel, Greece) close to Turkey, the recent appeared species on the coast with specimens/colonies higher than 2 were also categorized as established alien species. Among the established aliens, the species that affect biodiversity, human health and socio-economy are categorized as invasive alien species. In addition, some species with no definite evidence of their native or introduced status are considered as cryptogenic according to [27]. In the classification of the pathways for the introductions of alien species to Turkey, only primary pathways are considered.

In order to assess the distribution of alien species along the coasts of Turkey, the coasts were divided into grids with squares of 15 × 15 km. All data for the species distribution were extracted from respective papers and then entered to an Excel file, and then imported and digitized with ArcGIS 9.3 software. The coordinate system used in the Arcgis v9.3 software is WGS84.

## Results and discussion

### New knowledge on alien diversity in Turkey

**Fig 1:**
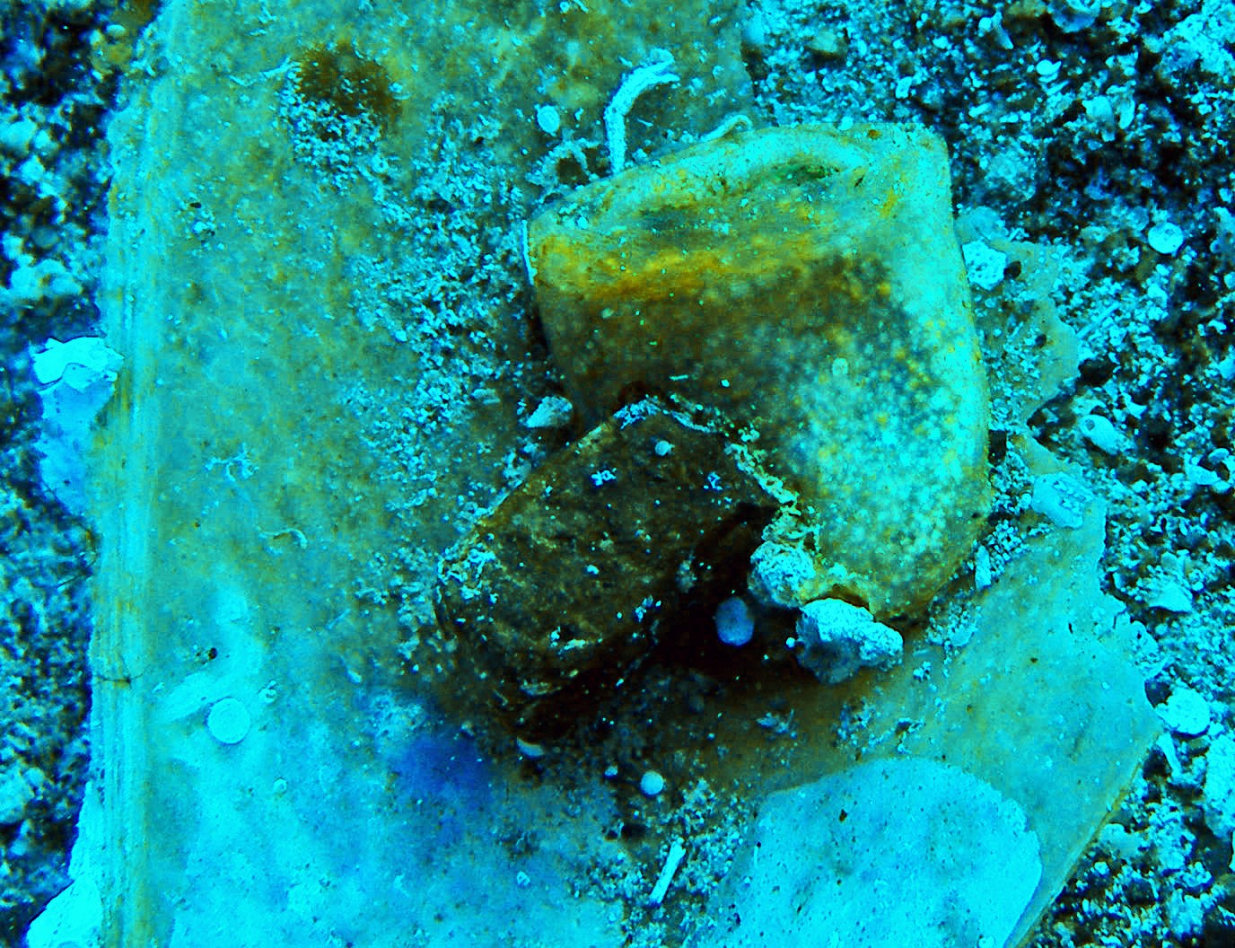
Underwater photograph of *Rhodosoma turcicum* observed on a dead *Pinna nobilis* shell in Fethiye Bay (Levantine Sea). Photograph by Melih Ertan ÇINAR.

In the present paper, two specimens (around 5 cm high) of the ascidian *Rhodosoma turcicum* are being reported for the first time from the coasts of Turkey (Levantine Sea), in Fethiye Bay (36°42’46.02’’N-28°54’29.91’’E) at 6 m depth on a dead shell of *Pinna nobilis* in 2008 (Fig 1). This species was originally described from the Red Sea on a madreporarian coral [28] and is widely distributed in tropical and some temperate areas (for the distribution, see [29]). This species was firstly reported from the Mediterranean in Lebanon in 1999 [30], and subsequently in Israel in 2004 [31]. The report of this species in Greece was unverified and excluded from the alien list of the country [32]. The main distinguishing character of the species is the horizontal fold of the body that acts as a lid over the apertures (Fig 1).

The present study also includes yet unpublished dataon range extensions (to different sea) of some alien species (personal observations by experts): the red alga *Polysiphonia morrowii* was found for the first time in the Aegean Sea (Saroz Bay) in 2018; the polychaetes *Leodice antennata* and *Dorvillea similis* in the Aegean Sea (Ildir Bay) in 2011; the bivalves *Arcuatula perfragilis* and *Paratapes textilis* in the Aegean Sea (Akbük) in 2018, and the fish *Paranthias furcifer* in the Levantine Sea (Antalya Bay) in 2003. The latter species was observed for almost a period of three months at the same reef in Üçadalar region and was photographed during scuba dives, but the finding remained unpublished until now since a specimen could not be captured.

### The actual number of alien species on the coasts of Turkey

The 2020’s update of marine alien species along the coasts of Turkey included 539 species belonging to 18 taxonomic groups, of which 404 species have become established in the regions and 135 species are casual (Table 1).

**Table 1:** The list of alien species and their first year of observations from the coasts of Turkey.

GROUPS/Species	BS	SM	AS	LS	ES	O	PW	H	DR
**RHODOPHYTA**	** **	** **	** **	** **	** **	** **	** **	** **	
*Asparagopsis armata* Harvey, 1855	1973	1984	1973	1968	**Inv**	Unk	S	Hs	I
*Asparagopsis taxiformis* (Delile) Trevisan de Saint-Léon, 1845		1984	2001	2005	**Inv**	RS	Co	Hs	I
*Acanthophora nayadiformis* (Delile) Papenfuss, 1968		1970	1845	1989	E	RS	Co	Hs	I,II
*Ganonema farinosum* (Lamouroux) Fan & Wang, 1974	1992	1899	1972	1989	E	RS	Co	Hs/Ss	I
*Bonnemaisonia hamifera* Hariot, 1891		1984	1998	1989	**Inv**	IP	?S	Hs	I
*Botryocladia madagascariensis* G.Feldmann, 1945			2005	1995	E	Unk	S	Hs	I
*Chondria curvilineata* F.S. Collins & Hervey, 1917 [as *Chondria collinsiana*]		1984			E	AT	S	Hs	II
*Colaconema codicola* (Børgesen) H.Stegenga, J.J.Bolton, & R.J.Anderson, 1997	1994	1984	1990	1988	E	Unk	S	Hs/Ss	I
*Griffithsia corallinoides* (Linnaeus) Trevisan, 1845		1986			E	Unk	S	Hs	I
*Hypnea spinella* (C. Agardh) Kützing, 1847			1983	1989	E	CT	S	Hs	I
*Lophocladia lallemandii* (Montagne) Schmitz, 1893			1970	1986	E	RS	Co	Hs	I,II
*Polysiphonia kampsaxiii* Boergesen, 1939		2015	1977	1989	E	IP	S	Hs	I
*Polysiphonia morrowii* Harvey, 1857		2007	2018		**Inv**	PO	S	Hs	I
*Polysiphonia paniculata* Montagne, 1842	1972		2001	1998	E	Unk	S	Hs	I
*Vertebrata fucoides* (Hudson) Kuntze 1891	1973	1986	1973	1997	E	Unk	S	Hs	I,II
*Rhodophysema georgei* Batters, 1900		1984			C	Unk	?S	Hs	I
*Gayliella fimbriata* (Setchell & N.L.Gardner) T.O.Cho & S.M.Boo, 2008				2014	C	IP	S	Hs	I
*Antithamnion hubbsii* E.Y.Dawson, 1962		2015			C	IP	S	Hs	I
*Grateloupia turuturu* Yamada, 1941		2015			**Inv**	IP	S	Hs	I
*Galaxaura rugosa* (J.Ellis & Solander) J.V.Lamouroux, 1816				2016	**Inv**	RS	Co	Hs	I
*Ceramium camouii* E.Y.Dawson, 1944		2018			C	IP	S	?	I
**OCHROPHYTA**									
*Cladosiphon zosterae* (J.Agardh) Kylin, 1940		1984	1983	1989	E	AT	S	Hs	I
*Chorda filum* (Linnaeus) Stackhouse 1797		1984			C	Unk	?S	Hs	I,II
*Colpomenia peregrina* Sauvageau, 1927		1998			E	IP	S	Hs	I
*Halothrix lumbricalis* (Kützing) Reinke, 1888	1998	1984	1980	1993	E	Unk	S	Hs	I
*Pylaiella littoralis* (Linnaeus) Kjellman, 1872	1984	1984	1983	1983	E	Unk	S	Hs	I
*Punctaria tenuissima* (C.Agardh) Greville, 1830	1993				E	AT	S	Hs	I
*Sphaerotrichia firma* (Gepp) A.D.Zinova, 1940		1984	1970	1984	E	Unk	S	Hs	I
*Stypopodium schimperi* (Buchinger ex Kützing) Verlaque & Boudouresque, 1991			1989	1991	**Inv**	RS	?Co	Hs	I
*Botrytella parva* (Takamatsu) H.-S.Kim, 1996		2006			C	IP	S	Hs	I
*Corynophlaea crispa* (Harv.) Kuckuck, 1929			2003		C	AT	S	Hs	I
*Scytosiphon dotyi* M.J.Wynne, 1969		2011			E	IP	Aq	Hs	I
*Ulonema rhizophorum* Foslie, 1894		2012			C	AT	S	Hs	I
*Dictyota cyanoloma* Tronholm, De Clerck, Gomez Garreta & Rull Lluch, 2010			2012		E	ST	S	Hs	I
*Cutleria multifida* (Turner) Greville, 1830	1976	1984	1970	1997	E	IP	Aq	Hs	I
*Microspongium globosum* Reinke 1888		2003			C	AT	S	Hs	I
**CHLOROPHYTA**									
*Caulerpa mexicana* Sonder ex Kützing, 1849				2007	E	IP	S	Ss	I
*Caulerpa cylindracea* Sonder, 1845			1993	2005	**Inv**	RS	Co	Hs/Ss	I,II
*Caulerpa racemosa* var. *lamourouxii* f. *requienii* (Montagne) Weber-van Bosse, 1898			1998	1980	E	RS	Co	Hs/Ss	I,II
*Caulerpa scalpelliformis* (R.Brown ex Turner) C. Agardh, 1817				1995	E	RS	Co	Ss	I,II
*Caulerpa taxifolia* var. *distichophylla* (Sonder) Verlaque, Huisman&Procacin, 2013			2010	2006	**Inv**	PO	S	Hs/Ss	I,II
*Codium fragile* subsp. *fragile* (Suringar) Hariot, 1889	1998	1998	1983	1998	**Inv**	Unk	S	Hs	I
*Codium taylorii* P.C. Silva, 1960			2011		E	IP	S	Hs	I
*Codium parvulum* (Bory *ex* Audouin) P.C.Silva, 2003			2012		E	RS	Co	Hs	I
*Pseudocodium okinawense* E.J.Faye, M.Uchimura & S.Smimada, 2008				2017	C	PO	S	Ss	III
*Ulva australis* Areschoug, 1854		2015			C	IP	Aq	Hs	I
**SPERMATOPHYTA**									
*Halophila stipulacea* (Forsskål) Ascherson, 1867			1956	1967	**Inv**	RS	Co	Ss	II
**FORAMINIFERA**					** **				
*Adelosina colomii* (Le Calvez & Le Calvez, 1958)			2003		C	NA	S	Hs/Ss	II,III
*Adelosina longirostra* (d’Orbigny, 1826)		2002			C	Unk	S	Hs/Ss	II-V
*Adelosina milletti* Wiesner, 1923		2002			E	PO	S	Hs/Ss	?
*Ammodiscus gullmarensis* Höglund, 1948			2003		C	Unk	S	Hs/Ss	II-VI
*Amphisorus hemprichii* Ehrenberg, 1840			2004	2002	**Inv**	Unk	?	Hs/Ss	I,II
*Amphistegina lessonii* d’Orbigny in Guérin-Méneville, 1832		2013	2007	2008	E	Unk	?	Hs/Ss	I,II
*Amphistegina lobifera* Larsen, 1976		2004	2001	1997	**Inv**	RS	Co	Hs/Ss	I-III
*Articulina alticostata* Cushman, 1944			2004	2001	E	PO	S	Hs/Ss	II
*Articulina carinata* Wiesner, 1923			1999		E	PO	S	Hs/Ss	I,II
*Articulina mayori* Cushman, 1922				1988	C	AT	S	Hs/Ss	III,IV
*Aschemonella aspera* Gooday and Holzmann, 2017		2010			C	PO	S	Hs/Ss	III
*Astacolus insolitus* (Schwager, 1866)		2008	2004		E	PO	S	Hs/Ss	II-VI
*Bolivina arta* MacFadyen, 1931			2003		C	PO	S	Hs/Ss	III-VI
*Bolivina striatula* Cushman, 1922		1995	2003	1988	E	Unk	?	Hs/Ss	II
*Brizalina simpsoni* (Heron-Allen and Earland, 1915)			2007		E	RS	Co	Hs/Ss	II
*Bulimina biserialis* Millett, 1900				1988	C	RS	Co	Hs/Ss	II,III
*Bulimina denudata* Cushman & Parker, 1938		2002			C	PO	S	Hs/Ss	II-V
*Candeina nitida* d’Orbigny, 1839		2011			C	Unk	S	Hs/Ss	VII
*Clavulina* cf. *multicamerata* Chapman, 1907				2002	E	RS	Co	Hs/Ss	II
*Cornuspiroides striolata* (Brady)		2008	2014		E	Unk	S	Hs/Ss	III
*Coscinospira acicularis* (Batsch, 1791)			2007		C	AT	S	Hs/Ss	I
*Cushmanina striatopunctata* (Parker and Jones, 1865)		2007	2002		C	Unk	S	Hs/Ss	II
*Cyclorbiculina compressa* (d’Orbigny, 1839)				2002	C	Unk	?	Hs/Ss	II
*Cymbaloporetta plana* (Cushman, 1915)		2011	2007	2002	E	RS	Co	Hs/Ss	I-III
*Cymbaloporetta squammosa* (d’Orbigny, 1839)			2012	2002	E	Unk	?	Hs/Ss	I,II
*Dendronina arborescen*s Heron-Allen and Earland, 1922		2012			C	Unk	S	Hs/Ss	II
*Dentalina albatrossi* (Cushman, 1923)		2002	2004		E	Unk	?	Hs/Ss	III
*Dentalina vertebralis* (Batsch, 1791)		2008			E	PO	S	Hs/Ss	III,IV
*Discogypsina vesicularis* A. Silvestri, 1937		2011			C	Unk	S	Hs/Ss	III
*Elphidium striatopunctatum* (Fichtel and Moll, 1798)				1997	C	RS	Co	Hs/Ss	II-IV
*Entosigmomorphina* sp.				2002	C	PO	S	Hs/Ss	II
*Euthymonacha polita* (Chapman, 1904)			2007		E	Unk	S	Hs/Ss	II
*Faujasina carinata* d’Orbigny, 1839		2008			C	NA	S	Hs/Ss	III,IV
*Fissurina faba* (Balkwill & Millett, 1884)		2002			C	NA	S	Hs/Ss	IV
*Globobulimina auriculata* (Bailey, 1894)			2003		C	Unk	S	Hs/Ss	?
*Guttulina ovata* (d’Orbigny, 1846)		2011			C	Unk	S	Hs/Ss	II,III
*Guttulina yabei* Cushman and Ozawa, 1929		2011			C	PO	S	Hs/Ss	II
*Haddonia* sp.			2009	2002	E	RS	Co	Hs/Ss	I,II
*Hansenisca soldanii* (d’Orbigny, 1826)				2008	C	RS	Co	Hs/Ss	I
*Hauerina diversa* Cushman, 1946			2007	1997	E	RS	Co	Hs/Ss	I,II
*Haynesina paucilocula* (Cushman, 1944)		2005			E	NA	S	Hs/Ss	III-V
*Heterocyclina tuberculata* (Möbius, 1880)				1988	E	RS	Co	Hs/Ss	II
*Heterostegina depressa* d’Orbigny, 1826				1988	E	RS	Co	Hs/Ss	I,II
*Iridia diaphan*a Heron-Allen and Earland, 1914			2006		E	PO	S	Hs/Ss	I
*Laevidentalina filiformis* (d’Orbigny, 1826)		2011			C	Unk	S	Hs/Ss	III
*Marginulina gummi* Saidova, 1975		2005			C	PO	S	Hs/Ss	III
*Marsipella elongata* Norman, 1878		2012			C	Unk	S	Hs/Ss	II
*Melonis affinis* (Reuss, 1851)		1995	2003		E	NA	S	Hs/Ss	II-VI
*Miliolinella cf*. *hybrida* (Terquem, 1878)				2002	C	RS	Co	Hs/Ss	II
*Nodobaculariella cristobalensis* McCulloch, 1977			2014		E	PO	S	Hs/Ss	I,II
*Nodobaculariella galapagosensis* McCulloch, 1977			2014		C	PO	S	Hs/Ss	II
*Nodophthalmidium antillarum* (Cushman, 1922)			2002	1995	E	RS	Co	Hs/Ss	I,II
*Nonion fabum* (Fichtel and Moll, 1798)		2011			C	AT	S	Hs/Ss	III
*Nonion subturgidum* (Cushman, 1924)		2005			C	IP	S	Hs/Ss	III
*Peneroplis arietinus* (Batsch, 1791)			2008	2002	E	RS	Co	Hs/Ss	I,II
*Peneroplis pertusus* (Forsskål in Niebuhr, 1775)		1998	1999	1988	E	RS	Co	Hs/Ss	I,II
*Peneroplis planatus* (Fichtel & Moll, 1798)		2013	1999	1988	C	RS	Co	Hs/Ss	I,II
*Planispirinella exigua* (Brady, 1879)				1988	C	RS	Co	Hs/Ss	II-IV
*Planogypsina acervalis* (Brady, 1884)			2002	2002	E	RS	Co	Hs/Ss	I,II
*Planogypsina squamiformis* (Chapman, 1901)			2001	2002	E	RS	Co	Hs/Ss	I,II
*Polymorphina fistulosa* Williamson, 1858		2015	2012		E	RS	Co	Hs/Ss	II,III
*Procerolagena gracilis* (Williamson, 1848)		2008			C	Unk	S	Hs/Ss	IV-VII
*Pseudoclavulina humilis* (Brady, 1884)		2012			C	Unk	S	Hs/Ss	VII
*Pseudomassilina australis* (Cushman, 1932)				1988	C	RS	Co	Hs/Ss	II
*Pseudomassilina reticulata* (Heron-Allen and Earland, 1915)				1988	C	RS	Co	Hs/Ss	I,II
*Pseudonodosaria brevis* (d’Orbigny, 1846)			2014		C	PO	S	Hs/Ss	III
*Pulleniatina obliquiloculata* (Parker & Jones, 1862)			2004		C	PO	S	Hs/Ss	II,III
*Pyramidulina catesbyi* (d’Orbigny, 1839)			2008	1988	E	RS	Co	Hs/Ss	II,III
*Pyramidulina perversa* (Schwager, 1866)			2002	2002	E	PO	S	Hs/Ss	II
*Pyrgo denticulata* (Brady, 1917)				2002	E	Unk	?	Hs/Ss	II
*Quinqueloculina carinatastriata* (Wiesner, 1923)		2002	2003		E	PO	S	Hs/Ss	II-V
*Quinqueloculina* cf. *mosharrafai* Said, 1949				2002	C	RS	Co	Hs/Ss	II
*Quinqueloculina* sp. C			2009		C	RS	Co	Hs/Ss	II
*Recurvoidella bradyi* (Robertson, 1891)			2003		C	Unk	S	Hs/Ss	III-VII
*Schlumbergerina alveoliniformis* (Brady, 1879)				2002	E	RS	Co	Hs/Ss	I,II
*Sigmoihauerina bradyi* (Cushman, 1917)				1988	C	Unk	?	Hs/Ss	?
*Siphonaperta arenata* (Said, 1949)		2002			C	Unk	?	Hs/Ss	III
*Siphonina tubulosa* Cushman, 1924		2005		2008	C	Unk	S	Hs/Ss	I
*Sorites orbiculus* Ehrenberg, 1839	2010		2001	1988	E	Unk	?	Hs/Ss	I,II
*Sorites variabilis* Lacroix, 1941			2007	2002	E	RS	Co	Hs/Ss	I,II
*Spiroloculina angulata* Cushman, 1917		2004	2006	1988	E	RS	Co	Hs/Ss	I-III
*Spiroloculina subcommunis* McCulloch, 1981		2008			C	AT	S	Hs/Ss	III-VII
*Stainforthia concava* (Hoeglund, 1947)		2008			C	Unk	S	Hs/Ss	IV
*Stainforthia fusiformis* (Williamson, 1848)		2008			C	Unk	S	Hs/Ss	VI,VII
*Textularia cushmani* Said, 1949		2002			C	IP	S	Hs/Ss	IV
*Triloculina affinis* d’Orbigny, 1852			2009		C	Unk	S	Hs/Ss	II
*Triloculina cf*. *fichteliana* d’Orbigny, 1839			2008	2004	E	RS	Co	Hs/Ss	I,II
*Triloculina* sp. A			2009		C	RS	Co	Hs/Ss	II
*Triloculinella asymmetrica* (Said, 1949)				1988	C	RS	Co	Hs/Ss	II,III
*Vaginulinopsis sublegumen* Parr, 1950			2004		E	PO	S	Hs/Ss	II
*Veleroninoides scitulus* (Brady, 1881)			2003		C	Unk	S	Hs/Ss	III-VII
**PORIFERA**									
*Paraleucilla magna* Klautau, Monteiro & Borojevic, 2004		2012	2004		**Inv**	WA	S	Hs	I
*Niphates toxifera *Vacelet, Bitar, Carteron, Zibrowius & Pérez, 2007				2019	E	?RS	?Co	Hs	I
**CNIDARIA**									
*Aequorea globosa* Eschscholtz, 1829				2011	E	RS	Co	P	I,II
*Aequorea vitrina* Gosse, 1853		2015			E	EA	S	P	I
*Cordylophora caspia* (Pallas, 1771)			2004		E	Unk	S	Hs	I
*Coryne eximia* Allman, 1859		1950			E	CT	S	Hs	I
*Clytia linearis* (Thorneley, 1900)			1977		E	RS	Co	Hs	I
*Eudendrium merulum* Watson, 1985		1953	1977		E	CT	S	Hs	I
*Filellum serratum* (Clarke, 1879)		1980	1977		E	CT	S	Hs	II
*Macrorhynchia philippina* Kirchenpauer, 1872				2005	**Inv**	RS	Co	Hs	I
*Sertularia marginata* (Kirchenpauer, 1864)			1977		E	CT	S	Hs	I
*Cassiopea andromeda* (Forsskål, 1775)			2011	2000	**Inv**	RS	Co	P	I
*Chrysaora pseudoocellata* Mutlu, Çağatay, Olguner & Yılmaz, 2020				2018	E	?RS	?Co	P	I
*Phyllorhiza punctata* von Lendenfeld, 1884			2011	2010	E	RS	Co	P	I
*Rhopilema nomadica* Galil, Spanier & Ferguson, 1990			2011	1995	**Inv**	RS	Co	P	I
*Marivagia stellata* Galil and Gershwin, 2010				2019	E	RS	Co	P	I
*Sagartiogeton laceratus* (Dalyell, 1848)		1998			E	EA	S	Hs	II
*Pachycerianthus multiplicatus* Carlgren, 1912				2009	E	EA	S	Ss	I
*Diadumene lineata* (Verrill, 1869)			1997		E	PO	S	Hs	I
*Diadumene cincta* Stephenson, 1925		2011			**Inv**	NA	S	Hs	I
**CTENOPHORA**					** **				
*Mnemiopsis leidyi* (Agassiz, 1865)	1993	1994	1994	1992	**Inv**	NA	S	P	I, II
*Beroe ovata* Mayer 1912	1996	2004			**Inv**	NA	S	P	I, II
**SIPUNCULA**					** **				
*Aspidosiphon* (*A*.) *elegans* (Chamisso & Eysenhardt, 1821)			2006	2005	E	RS	Co	Hs	I
*Nephasoma (Nephasoma) eremita* (Sars, 1851)				2005	C	?PO	S	Ss	III
**POLYCHAETA**									
*Lepidonotus tenuisetosus* (Gravier, 1902)				2005	E	RS	Co	Hs	I
*Pisione guanche* San Martín, López & Núñez, 1999				2005	E	AT	S	Ss	I
*Phyllodoce longifrons* Ben-Eliahu, 1972				2011	E	RS	Co	Hs/Ss	I
*Eurythoe complanata* (Pallas, 1766)				1993	**Inv**	?RS	?Co	Hs	I
*Linopherus canariensis* Langerhans, 1881			2005	1993	E	AT	S	Hs/Ss	I
*Eusyllis kupfferi* Langerhans, 1879				2005	E	?AT	S	Hs	I
*Exogone africana* (Hartmann-Schröder, 1974)				2011	E	RS	Co	Ss	II
*Exogone breviantennata* Hartmann-Schröder, 1959				2005	E	RS	Co	Hs	I
*Prosphaerosyllis longipapillata* (Hartmann-Schröder, 1979)			2004	2005	E	PO	S	Ss	II
*Syllis ergeni* Çinar, 2005			2004	2005	**Inv**	RS	Co	Hs	I
*Syllis pectinans* Haswell, 1920			2004		E	PO	S	Hs	I
*Ceratonereis mirabilis* Kinberg, 1866			2011	2005	E	RS	Co	Hs/Ss	I,II,III
*Leonnates decipiens* Fauvel, 1929				2005	E	RS	Co	Hs	I
*Leonnates indicus* Kinberg, 1866				2005	**Inv**	RS	Co	Hs	I
*Leonnates persicus* Wesenberg-Lund, 1949			2001	2000	E	RS	Co	Ss	II-IV
*Nereis persica* Fauvel, 1911		1959		2005	E	RS	Co	Ss	I,II,III
*Nereis jacksoni* Kinberg, 1866				2005	E	RS	Co	Ss	I,II
*Pseudonereis anomala* Gravier, 1900			2004	1973	**Inv**	RS	Co	Hs	I
*Glycinde bonhourei* Gravier, 1904			2009	2005	E	RS	Co	Ss	I
*Diopatra marocensis* Paxton, Fadlaoui & Lechapt, 1995			2005	2005	E	EA	S	Ss	I,II
*Lumbrineris perkinsi* Carrera-Parra, 2001				2005	E	RS	?Co	Hs	I,II
*Leodice antennata* (Savigny, 1820)			2011	1993	**Inv**	RS	Co	Hs	I
*Lysidice collaris* Grube, 1870			1993	1993	E	RS	Co	Hs/Ss	I,II
*Palola valida* (Gravier, 1900)				2005	E	RS	Co	Hs	I
*Dorvillea similis* (Crossland, 1924)			2011	2005	**Inv**	RS	Co	Hs/Ss	I,II
*Aricidea bulbosa* Hartley, 1984		2013	2017		E	RS	Co	Ss	I-III
*Laonice norgensis* Sikorski, 2003			2000		C	AT	S	Ss	IV
*Polydora cornuta* Bosc, 1802	2012	2002	1986		**Inv**	WA	S	Ss	I,II
*Prionospio* (*Aquilaspio*) *krusadensis* Fauvel 1929				2005	E	IP	S	Ss	I
*Prionospio* (*Aquilaspio*) *sexoculata* Augener, 1918				2005	E	IP	S	Ss	I
*Prionospio* (*Prionospio*) *depauperata* Imajima, 1990			2000	2005	**Inv**	PO	S	Ss	I,II
*Prionospio* (*Prionospio*) *paucipinnulata* Blake & Kudenov, 1978			2000	2005	E	PO	S	Ss	I,II
*Prionospio* (*Prionospio*) *saccifera* Mackie & Hartley, 1990			2000	1995	E	RS	Co	Ss	I-III
*Prionospio* (*Minuspio*) *pulchra* Imajima 1990	2000	2008	2000	2005	**Inv**	IP	S	Ss	I,II
*Pseudopolydora paucibranchiata* Okuda, 1937		2008	2000	2005	**Inv**	IP	S	Hs/Ss	I
*Spiophanes algidus* Meißner, 2005			2000		C	IO	S	Ss	IV
*Streblospio gynobranchiata* Rice & Levin, 1998		2005	2000		**Inv**	WA	S	Ss	I,II
*Neopseudocapitella brasiliensis* Rullier & Amoureux, 1979			2001		E	Unk	S	Ss	II
*Notomastus aberans* Day, 1957		2013	1980	2000	E	RS	Co	Ss	I-III
*Notomastus mossambicus* (Thomassin, 1970)				2005	**Inv**	RS	Co	Ss	I-III
*Chaetozone corona* Berkeley & Berkeley, 1941		2010	1980	2005	E	?PO	S	Ss	I,II
*Timarete caribous* (Grube, 1859)				2005	C	WA	S	Hs	I
*Timarete punctata* (Grube, 1859)				2005	E	RS	Co	Hs	I
*Semiodera cinari* Salazar-Vallejo, 2012				2005	E	IP	S	Hs	I
*Stylarioides grubei* Salazar-Vallejo, 2011				2005	E	IO	S	Hs	I
*Metasychis gotoi* (Izuka, 1902)		2008	1996	2000	E	RS	Co	Ss	II,III
*Pista unibranchia* Day, 1963			1998	1993	E	RS	Co	Ss	I,II
*Loimia medusa* (Savigny, 1818)		1959		2005	E	RS	?Co	Ss	?
*Polycirrus twisti* Potts, 1928				2005	E	RS	Co	Hs/Ss	I
*Branchiomma bairdi* (McIntosh, 1885)				2005	**Inv**	Unk	?S	Hs/Ss	I
*Branchiomma luctuosum* Grube, 1869				2005	**Inv**	RS	Co	Hs	I
*Desdemona ornata* Banse, 1957		2005			E	IP	S	Ss	I
*Laonome triangularis* Hutchings & Murray, 1984				2005	E	PO	S	Ss	I
*Ficopomatus enigmaticus* (Fauvel, 1923)		1952	1972		**Inv**	ST	S	Hs	I
*Hydroides brachyacanthus* Rioja, 1941				2005	**Inv**	IP	?S	Hs	I
*Hydroides diramphus* Mörch, 1863		1894		2005	**Inv**	CT	S	Hs	I
*Hydroides elegans* (Haswell, 1883)		2012	1972	1991	**Inv**	CT	S	Hs	I
*Hydroides heterocerus* (Grube, 1868)				2005	E	RS	Co	Hs	I,II
*Hydroides homoceros* Pixell, 1913				2005	E	RS	Co	Hs	I
*Hydroides minax* (Grube, 1878)				2005	E	RS	Co	Hs	I
*Hydroides operculata* (Treadwell, 1929)				2005	**Inv**	IO	S	Hs	I
*Spirobranchus kraussii* (Baird, 1865)				2005	**Inv**	RS	Co	Hs	I
*Spirobranchus tetraceros* (Schmarda, 1861)				2005	E	RS	Co	Hs	I,II
*Janua* (*Dexiospira*) *steueri* (Sterzinger, 1909)				2005	E	RS	Co	Ss	I,II
*Spirorbis marioni* Caullery & Mesnil, 1897			1987	2005	E	PO	S	Hs	I
**ARTHROPODA**									
*Anoplodactylus californicus* Hall, 1912				1959	E	RS	Co	Hs	I
*Ammothea hilgendorfi* (Böhm, 1879)				2010	E	PO	S	Hs	I
*Amphibalanus eburneus* (Gould, 1841)	1968	1939	1968	1968	**Inv**	AT	S	Hs	I
*Amphibalanus improvisus* (Darwin, 1854)	1988	1892	1968	1968	**Inv**	WA	S	Hs	I
*Balanus trigonus* Darwin, 1854			1993		E	CT	S	Hs	I
*Heterosaccus dollfusi* Boschma, 1960				1994	**Inv**	RS	Co	Pz	I,II
*Pleopis schmackeri* (Poppe, 1889)			2017	2012	E	IP	Co/S	P	I,II
*Acartia tonsa* Dana, 1848		1993	2001	1998	E	Unk	S	P	I
*Acrocalanus gibber* Giesbrecht, 1888		1998		1999	E	ST	S	P	I,II
*Calanopia elliptica* (Dana, 1846)				1999	E	RS	Co	P	I
*Caligus lagocephali* Pillai, 1961				2011	C	IP	?	Pz	I,II
*Centropages furcatus* (Dana, 1849)		1998		1999	E	RS	Co	P	I-III
*Dioithona oculata* (Farran, 1913)				2013	E	IP	S	P	I,II
*Mitrapus oblongus* (Pillai, 1964)				2010	E	RS	Co	Pz	?
*Labidocera pavo* Giesbrecht, 1889				1999	E	RS	Co	P	I
*Lernanthropus callionymicola* El-Rashidy & Boxshall, 2012				2013	E	RS	Co	Pz	?
*Oithona davisae* Ferrari and Orsi, 1984	2009	2014	2017	2018	**Inv**	PO	S	P	I,II
*Paracartia grani* Sars G.O., 1904		1998			E	AT	S	P	I,II
*Parvocalanus crassirostris* (Dahl F., 1894)		1998		1998	E	RS	Co	P	I,II
*Parvocalanus elegans* Andronov, 1972		1998		1998	E	RS	Co	P	I-III
*Parvocalanus latus* Andronov, 1972		1998		1998	E	?RS	?Co	P	I-III
*Taeniacanthus lagocephali* Pearse, 1952				2011	C	IP	?	Pz	I,II
*Clorida albolitura* Ahyong & Naiyanetr, 2000				2009	E	RS	Co	Ss	II
*Cloridina cf*. *ichneumon* (Fabricius, 1798)				2019	C	RS	Co	Ss	II
*Erugosquilla massavensis* (Kossmann, 1880)		2002	1987	1959	**Inv**	RS	Co	Ss	I-IV
*Ampithoe bizseli* Özaydınlı and Coleman, 2012			2010		E	IP	S	Hs	I
*Caprella scaura* Templeton, 1836		2012	2008		E	IP	S	Hs	I
*Latigammaropsis togoensis* (Schellenberg,1925)				2005	E	Unk	?S	Hs	I
*Linguimaera caesaris* Krapp‐Schickel, 2003			1976	1976	E	RS	Co	Hs	I
*Paracerceis sculpta* Holmes,1904				2015	C	IP	S	Hs	I
*Paradella dianae* Menzies,1962			2004	2008	E	Unk	?S	Hs/Ss	I
*Sphaeroma walkeri* (Stebbing, 1905)			1995	2015	E	RS	Co	Hs	I
*Paradoxapseudes intermedius* (Hansen, 1895)		2006	1972		E	AT	?S	Hs/Ss	I,II
*Eocuma sarsii* (Kossmann, 1880)			1976		E	RS	Co	Hs/Ss	I,II
*Actaea savignii* (H. Milne Edwards, 1834)				2011	E	RS	Co	Hs/Ss	I
*Alpheus lobidens* De Haan, 1849			2014	1969	E	RS	Co	Ss	I
*Alpheus migrans* Lewinsohn & Holthuis, 1978				1993	E	RS	Co	Ss	I-III
*Alpheus rapacida* de Man, 1908			2005	1981	E	RS	Co	Ss	I-III
*Arcania brevifrons* Chen 1989			2019		C	RS	Co	Ss	II
*Atergatis roseus* (Rüppell, 1830)			2004	1987	E	RS	Co	Hs	I,II
*Calappa hepatica* (Linnaeus, 1758)				1992	C	RS	Co	Ss	I-III
*Callinectes sapidus* Rathbun, 1896	2013	2001	1967	1959	**Inv**	WA	S	Ss	I,II
*Carupa tenuipes* Dana, 1851			2003	1996	E	RS	Co	Hs	I,II
*Charybdis hellerii* (Milne Edwards, 1867)			2003	1987	**Inv**	RS	Co	Hs/Ss	I,II
*Charybdis longicollis* Leene, 1938			2002	1959	**Inv**	RS	Co	Ss	I-IV
*Coleusia signata* (Paulson, 1875)		2006		1976	E	RS	Co	Hs/Ss	I,II
*Daira perlata* (Herbst, 1790)				1988	C	RS	Co	Hs/Ss	I,II
*Eucrate crenata* de Haan, 1835			2018	1987	E	RS	Co	Ss	I-IV
*Eurycarcinus integrifrons* De Man, 1879				2009	E	IO	S	Ss	II
*Gonioinfradens giardi* (Nobili, 1905)				2009	C	IP	S	Hs/Ss	I
*Ixa monodi* Holthuis & Gottlieb, 1956			2005	1955	E	RS	Co	Ss	I,II
*Leptochela pugnax* de Man, 1916			2000	1966	E	RS	Co	Ss	I-IV
*Macrophthalmus indicus* Davie, 2012			2000	1994	E	RS	Co	Ss	I-IV
*Metapenaeopsis aegyptia* Galil & Golani, 1990				2003	E	RS	Co	Ss	I
*Metapenaeopsis mogiensis consobrina* (Nobili, 1904)				2003	E	RS	Co	Ss	I
*Metapenaeus affinis* (H. Milne Edwards, 1837)			2008		E	RS	Co	Ss	I,II
*Metapenaeus monoceros* (Fabricius, 1798)				1959	**Inv**	RS	Co	Ss	I,II
*Metapenaeus stebbingi* (Nobili, 1904)				1966	**Inv**	RS	Co	Ss	I-III
*Micippa thalia* (Herbst, 1803)			2006	1994	E	RS	Co	Ss	I-IV
*Matuta victor* (Fabricius, 1781)			2017	2015	E	RS	Co	Ss	I,II
*Myra subgranulata* Kossmann, 1877				1930	E	RS	Co	Ss	I-IV
*Ogyrides mjoebergi* (Balss, 1921)				2005	E	RS	Co	Ss	I
*Palaemonella rotumana* (Borradaile, 1898)				1999	E	RS	Co	Hs	I,II
*Penaeus aztecus* Ives, 1891	2017		2015	2009	E	WA	S	Ss	I,II
*Penaeus hathor* (Burkenroad, 1959)			2006	2002	**Inv**	RS	Co	Ss	I,II
*Penaeus merguiensis* (De Man, 1888)				2006	C	IP	Aq	Ss	II
*Penaeus pulchricaudatus* Stebbing, 1914		2001	2001	1930	**Inv**	RS	Co	Ss	I-III
*Penaeus semisulcatus* de Haan, 1844				1930	**Inv**	RS	Co	Ss	I-IV
*Penaeus subtilis* (Pérez Farfante, 1967)				2012	C	AT	S	Ss	II
*Percnon gibbesi* (H. Milne Edwards, 1853)				2005	**Inv**	TA	S	Hs	I,II
*Pilumnopeus vauquelini* (Audouin,1826)				1966	E	RS	Co	Hs	I
*Pilumnus minutus* De Haan,1835			2000	2003	E	RS	Co	Hs	I
*Portunus segnis* (Forskål, 1775)			2004	1928	**Inv**	RS	Co	Ss	I-IV
*Processa macrodactyla* Holthuis, 1952			1995		E	TA	S	Ss	II
*Saron marmoratus* (Olivier, 1811)				2018	C	RS	Co	Hs	II
*Sicyonia lancifer* (Olivier, 1811)				2014	C	RS	Co	Ss	III
*Thalamita poissonii* (Audouin, 1826)			1981	1959	E	RS	Co	Ss	I,II
*Trachysalambria palaestinensis* Steinitz, 1932				1968	E	RS	Co	Ss	I-V
*Urocaridella pulchella* Yokes & Galil, 2006				2003	E	RS	Co	?	II
**MOLLUSCA**									
*Diodora ruppellii* (Sowerby I, G.B., 1835)				1988	E	RS	Co	Hs	I
*Trochus erithreus* Brocchi, 1821				1992	E	RS	Co	Hs	I
*Pseudominolia nedyma* (Melville, 1897)				1992	E	RS	Co	Ss	I,II
*Stomatella impertusa* (Burrow, 1815)				1999	C	RS	Co	Hs	I
*Nerita sanguinolenta* Menke, 1829				2004	E	RS	Co	Ss	I
*Smaragdia souverbiana* (Montrouzier in Souverbie & Montrouzier, 1863)				1993	E	RS	Co	Ss	I
*Cerithidium diplax* (Watson, R. B., 1886)				1986	**Inv**	PG	S	Ss	I,II
*Cerithidium perparvulum* (Watson, R. B., 1886)				1992	E	PO	S	Ss	I
*Cerithium scabridum* Philippi, 1848			1990	1986	**Inv**	RS	Co	Hs/Ss	I
*Rhinoclavis kochi* (Philippi, 1848)				1986	E	RS	Co	Ss	I
*Varicopeza pauxilla *(A. Adams, 1855)				2016	E	RS	Co	Ss	II,III
*Diala semistriata *(Philippi, 1849)				2002	E	RS	Co	Ss	I
*Gibborissoia virgata* (Philippi, 1849)				1997	E	RS	Co	Hs	I
*Finella pupoides* Adams, A., 1860			2001	1958	**Inv**	RS	Co	Ss	I
*Metaxia bacillum* (Issel, 1869)				1992	E	RS	Co	Hs	I
*Viriola bayani* Jousseaume, 1884			2017		E	RS	Co	Ss	I,II
*Cerithiopsis pulvis* (Issel, 1869)				1990	E	RS	Co	Ss	I
*Cerithiopsis tenthrenois* (Melvill, 1896)				1990	E	RS	Co	Ss	I
*Cycloscala hyalina* (Sowerby II, G. B., 1844)				1995	E	RS	Co	Ss	I
*Sticteulima lentiginosa* (Adams, A., 1861)				1989	E	RS	Co	Ss	I
*Rissoina ambigua* (Gould, 1849)				2003	C	RS	Co	Ss	II
*Rissoina bertholleti* Issel, 1869				1985	E	RS	Co	Ss	I
*Caecum sepimentum* de Folin, 1868				2013	C	RS	?Co	Ss	II
*Conomurex persicus* (Swainson, 1821)			1991	1978	**Inv**	PG	S	Hs/Ss	I,II
*Circulus novemcarinatus *(Melvill, 1906)				2010	E	RS	Co	Ss	I
*Crepidula fornicata* (Linnaeus, 1758)			2012		E	Unk	Aq/S	Ss	I,II
*Eratoena sulcifera *(Gray in Sowerby I, G. B., 1832)				2013	C	IP	?S	Hs	I
*Purpuradusta gracilis notata* (Gill, 1858)				1982	E	RS	Co	Hs	I
*Eunaticina papilla* Gmelin, 1791				2013	C	IP	S	Ss	I
*Ergalatax junionae* Houart, 2008			2002	1992	E	RS	S	Hs	I
*Rapana venosa* (Valenciennes, 1846)	1960	1993	1995		**Inv**	PO	S	Ss	I
*Indothais lacera *(Born, 1778)				1991	E	IO	S	Hs/Ss	I
*Crithe cossinea* T. Cossignani, 1997				2014	C	IP	S	Ss	II
*Zafra obesula* (Hervier, 1899)				2010	C	RS	?Co	Ss	II
*Zafra pumila* (Dunker, 1858)				2010	C	RS	?Co	Ss	II
*Zafra savignyi* (Moazzo, 1939)				1986	E	RS	Co	Ss	I,II
*Zafra selasphora* (Melvill & Standen, 1901)				1993	E	RS	Co	Ss	I
*Lienardia mighelsi* Iredale & Tomlin, 1917			2003		C	IP	Co/S	Ss	III
*Pseudorhaphitoma iodolabiata *(Hornung & Mermod, 1929)				2011	E	RS	Co	Ss	II
*Pyrgulina fischeri *Hornung & Mermod, 1925				1989	E	RS	Co	Ss	I
*Pyrgulina pupaeformis *(Souverbie, 1865)				1963	E	RS	Co	Hs/Ss	I
*Pyrgulina nana* Hornung & Mermod, 1924			2000	1997	C	RS	?S	Ss	I,II
*Pyrgulina pirinthella *Melvill, 1910				1989	E	RS	Co	Ss	I,II
*Cingulina isseli* (Tryon, 1886)				1986	E	RS	Co	Ss	I
*Iolaea neofelixoides* (Nomura, 1936)				1994	C	PO	?S	Ss	I
*Monotygma fulva* (Adams, A., 1853)			2000	2017	E	RS	Co	Ss	II
*Monotygma lauta* (Adams, A., 1853)			2014	1989	E	RS	Co	Ss	I,II
*Odostomia lorioli* (Hornung & Mermod, 1924)				2007	E	RS	Co	Ss	I,II
*Oscilla galilae *Bogi, Karhan & Yokeş, 2012				1992	C	IP	?S	?	I, II
*Syrnola cinctella* Adams, A., 1860				1994	C	RS	Co	Ss	I
*Syrnola fasciata* Jickeli, 1882			2001	1963	**Inv**	RS	Co	Ss	I
*Syrnola lendix* (Adams, A., 1853)				1988	E	IO	Co	Ss	I
*Turbonilla edgarii* (Melvill, 1896)				1989	E	RS	Co	Ss	?
*Amathina tricarinata* (Linnaeus, 1767)				2000	E	RS	Co	Hs	I
*Leucotina natalensis* Smith, E. A., 1910			2015	1986	E	RS	Co	Ss	I
*Bulla arabica* Malaquias & Reid, 2008			1998	2001	E	RS	?Co	Ss	II
*Pyrunculus fourierii* (Audouin, 1826)			2015	1989	**Inv**	RS	Co	Ss	II,III
*Retusa desgenettii* (Audouin, 1826)			2002		E	RS	Co	Ss	I,II
*Lamprohaminoea cyanomarginata* (Heller & Thompson, 1983)			2002	2002	E	RS	Co	Hs	II
*Biuve fulvipunctata* (Baba, 1938)				1959	E	RS	Co	Ss	I,II
*Acteocina crithodes (*Melvill & Standen, 1901)				2003	C	IP	S	Ss	II
*Acteocina mucronata* (Philippi, 1849)				1986	E	RS	Co	Ss	?
*Mnestia girardi* (Audouin, 1826)			1996	1990	E	RS	Co	Ss	II
*Oxynoe viridis* (Pease, 1861)				2002	E	IP	S	Ss	I
*Elysia grandifolia* Kelaart, 1858				2001	E	IO	S	Hs	I
*Elysia tomentosa* Jensen, 1997				2001	E	?IP	S	Hs	I,II
*Bursatella leachii* Blainville, 1817			1959	1959	E	RS	?Co	Hs	I
*Notarchus punctatus* Philippi, 1836			2004	2002	E	IP	S	Ss	I,II
*Syphonota geographica* (Adams, A. & Reeve, 1850)				1999	E	RS	Co	Ss	I,II
*Berthellina citrina* (Rüppell & Leuckart, 1828)				2005	E	RS	Co	Hs	I
*Chromodoris quadricolor* (Rüppell & Leuckart 1830)				2004	C	IO	S	Hs	II
*Goniobranchus annulatus *(Eliot, 1904)			2020	2008	E	RS	Co	Hs	I
*Goniobranchus obsoletus *(Rüppell & Leuckart, 1830)				2019	E	RS	Co	Hs	I
*Hypselodoris infucata* Rueppel & Leuckart, 1828				1999	E	RS	Co	Hs	I
*Dendrodoris fumata* (Rüppell & Leuckart, 1830)				2010	E	RS	Co	Hs	I
*Plocamopherus ocellatus* Rüppell & Leuckart, 1828				1998	E	RS	Co	Hs	I
*Plocamopherus tilesii* Bergh, 1877				2009	C	IP	S	Hs	III
*Melibe viridis* (Kelaart, 1858)				2000	E	IO	S	Hs	I
*Baeolidia moebii* Bergh, 1888				2007	C	RS	Co	Ss	II
*Coryphellina rubrolineata* O’Donoghue, 1929			2003	2001	E	RS	Co	Hs	I,II
*Siphonaria belcheri* Hanley, 1858				1999	E	IO	S	HS	I
*Siphonaria crenata* Blainville 1827				1999	E	RS	Co	Hs	I
*Anadara transversa* (Say, 1822)			1977		**Inv**	WA	S	Ss	I,II
*Anadara broughtonii* (Schrenck, 1867)				1998	C	IO	S	Ss	?
*Anadara kagoshimensis* (Tokunaga, 1906)	2003	1993	1995		E	IP	S	Ss	I,II
*Anadara natalensis* (Krauss, 1848)				1985	**Inv**	RS	Co	Ss	I,II
*Arcuatula perfragilis* (Dunker, 1857)			2018	2010	C	RS	S	Ss	I
*Arcuatula senhousia* (Benson, 1842)		2012	2012	2007	E	IP	?S	Ss	I
*Brachidontes pharaonis* (Fischer, P., 1870)			1990	1978	**Inv**	RS	Co	Hs	I
*Septifer bilocularis* (Linnaeus, 1758)				2006	C	RS	S	Hs	II
*Septifer cumingii* Récluz, 1849			2017	2001	E	RS	S	Hs	I
*Magallana gigas* (Thunberg, 1793)		2004	2006	1998	E	PO	Aq	Hs	I,II
*Saccostrea cuccullata* (Born, 1778)				1998	E	IP	S	Hs	I,II
*Dendostrea folium* (Linnaeus, 1758)			2015	1998	E	IP	?S	Hs	I
*Pinctada imbricata radiata* (Leach, 1814)			1987	1971	**Inv**	RS	Co	Hs	I
*Electroma vexillum* (Reeve, 1857)				2002	E	RS	Co	Hs	I
*Isognomon legumen* (Gmelin, 1791)			2017		E	RS	Co	Hs	I
*Malleus regula* (Forsskål in Niebuhr, 1775)			2004	1973	E	RS	Co	Hs	I
*Spondylus spinosus* Schreibers, 1793				1991	**Inv**	RS	Co	Hs	I,II
*Centrocardita akabana* (Sturany, 1899)				2005	C	RS	Co	Ss	I
*Chama asperella* Lamarck, 1819		1990	2006	2006	E	IP	?S	HS	I
*Chama pacifica* Broderip, 1835				1992	**Inv**	RS	Co	Hs	I,II
*Afrocardium richardi* (Audouin, 1826)				2000	E	RS	Co	Ss	?
*Fulvia fragilis* (Forsskål in Niebuhr, 1775)			2001	1986	**Inv**	RS	Co	Ss	I
*Psammacoma gubernaculum* (Hanley, 1844)				1992	C	WA	S	Ss	IV
*Nitidotellina valtonis* (Hanley, 1844)				1995	C	RS	Co	Ss	I
*Ervilia scaliola* Issel, 1869				2013	C	RS	Co	Ss	I,II
*Clementia papyracea* (Gmelin, 1791)				1985	E	RS	Co	Ss	III
*Gafrarium pectinatum* (Linnaeus, 1758)				1986	E	RS	Co	Ss	I
*Microcirce consternans* P. G. Oliver & Zuschin, 2001				2013	C	RS	Co	Ss	I
*Paratapes textilis* (Gmelin, 1791)			2018	1985	E	RS	Co	Ss	I,II
*Petricola fabagella Lamarck*, *1818*				1999	E	RS	Co	Hs	I
*Ruditapes philippinarum* (Adams & Reeve, 1850)		2004	2000		**Inv**	PO	Aq	Ss	I
*Timoclea roemeriana* (Issel, 1869)				2010	C	RS	Co	Ss	II
*Mya arenaria* Linnaeus, 1758	1998	1993	2008		E	WA	S	Ss	II
*Sphenia rueppelli* Adams, A., 1850				1998	E	RS	?Co	Hs	I
*Martesia striata* (Linnaeus, 1758)				2014	C	Unk	S	Hs	I
*Teredo bartschi* Clapp, 1923				2013	E	RS	Co	Hs	I
*Teredothyra dominicensis* (Bartsch, 1921)				2010	E	WA	S	Hs	II
*Cucurbitula cymbium* (Spengler, 1783)				1990	E	RS	Co	Ss	I-III
*Laternula anatina* (Linnaeus, 1758)				1992	E	RS	Co	Ss	I-III
*Amphioctopus aegina* (Gray, 1849)				1992	E	RS	Co	Ss	III
*Sepioteuthis lessoniana* d’Orbignyi, 1826				2002	**Inv**	RS	Co	P	I-III
**BRYOZOA**					** **				
*Amathia verticillata* (delle Chiaje, 1822)			2015		**Inv**	AT	S	Hs	I
*Celleporaria brunnea* (Hincks, 1884)			2004		**Inv**	AT	S	Hs	I
*Hippopodina* sp. A				2015	E	RS	Co	Hs	I
*Microporella coronata* (Audouin, 1826)				1992	E	RS	Co	Hs	II,III
*Parasmittina egyptiaca* (Waters, 1909)				2015	E	RS	Co	Hs	I
*Rhynchozoon larreyi* (Audouin, 1826)			1962		E	RS	Co	Ss	I
*Watersipora arcuata* Banta, 1969			2015	2015	E	PO	S	Hs	I
**ECHINODERMATA**									
*Asterias rubens* Linnaeus, 1758	2003	1993			**Inv**	AT	S	Hs	I,II
*Amphiodia* (*Amphispina*) *obtecta* Mortensen, 1940				2005	E	IP	S	Ss	I-III
*Ophiactis macrolepidota* Marktanner-Turneretscher, 1887				2005	C	RS	Co	Ss	I
*Ophiactis savignyi* (Müller & Troschel, 1842)			1993	2005	E	RS	Co	Hs	I
*Diadema setosum* (Leske, 1778)			2014	2006	**Inv**	RS	Co	Hs	I
*Holothuria* (*Theelothuria*) *hamata* Pearson, 1913				2017	E	RS	Co	Ss	II
*Synaptula reciprocans* (Forrskål, 1775)			2001	2005	**Inv**	RS	Co	Hs	I,II
**CHAETOGNATHA**					** **				
*Ferosagitta galerita* (Dallot, 1971)				2003	E	IO	S	P	I,II
**TUNICATA**									
*Ascidiella aspersa* (Müller, 1776)	1973	1884	1969	1973	E	NA	S	Hs	I
*Ciona robusta* Hoshino & Tokioka, 1967			2016		**Inv**	?IP	S	Hs	I
*Clavelina oblonga* Herdman, 1880			2015		E	WA	S	Hs	I
*Didemnum ahu* Monniot C. & Monniot F., 1987			2015		**E**	?CT	S	Hs	I
*Diplosoma listerianum* (Milne Edwards, 1841)		1894	1968	2015	E	?AT	S	Hs	I
*Microcosmus exasperatus* Heller, 1878			2004	2008	E	RS	Co	Hs	I
*Microcosmus squamiger* Michaelsen, 1927			2015		**E**	?ST	S	Hs	I
*Phallusia nigra* Savignyi, 1816			2011	2005	**Inv**	WA	?S	Hs	I,II
*Polyclinum constellatum* Savigny, 1816			2016		E	RS	Co	Hs	I
*Pyura* (= *Herdmania*) *momus* (Savigny, 1816)				2001	E	RS	Co	Hs	I,II
*Rhodosoma turcicum* (Savigny, 1816)				2008	E	CT	S	Hs	I
*Styela clava* Herdman, 1881		2012			**Inv**	PO	S	Hs	I
*Styela plicata* (Lesueur, 1823)			1973	1968	**Inv**	?AT	S	Hs	I
*Symplegma brakenhielmi* (Michaelsen, 1904)			2016	2005	**Inv**	RS	Co	Hs	I,II
**PISCES**									
*Himantura leoparda* (Manjaji, Matsumoto & Last, 2008)				2016	C	RS	Co	Ss	IV
*Himantura uarnak* (Forsskål, 1775)				1966	E	RS	Co	Ss	I,II
*Abudefduf cf*. *saxatilis/vaigiensis/troschelii*			2016		C	AT	?S	Hs	I
*Acanthopagrus bifasciatus* (Forsskål, 1775)			2018		C	RS	Co	Ss	I
*Alepes djedaba* (Forsskål, 1775)	2017		1966	1955	**Inv**	RS	Co	P	I-III
*Apogonichthyoides pharaonis* (Bellotti, 1874)			2002	1984	E	RS	Co	Hs/Ss	I,II
*Atherinomorus forskalii* (Rüppell, 1838)			1966	1949	**Inv**	RS	Co	P	I
*Bodianus speciosus* (Bowdich, 1825)			2018		C	TA	S?	Hs	II
*Bregmaceros nectabanus* Whitley, 1941			2005	2002	E	RS	Co	Ss	I,II
*Callionymus filamentosus* Valenciennes, 1837			2014	1983	E	RS	Co	Ss	I-III
*Champsodon nudivittis* (Ogilby, 1895)			2014	2008	**Inv**	RS	Co	Ss	I,II
*Chanos chanos* (Forsskål, 1775)				2011	C	RS	Co	P	III
*Cheilodipterus novemstriatus* (Rüppell, 1838)				2014	E	RS	Co	Hs/Ss	I,II
*Cyclichthys spilostylus* (Leis & Randall, 1982)				2011	C	RS	Co	Ss	III
*Cynoglossus sinusarabici* (Chabanaud, 1913)			2014	1955	E	RS	Co	Hs	I,II
*Decapterus russelli* (Rüppell, 1830				2009	E	RS	Co	P	I,II
*Diplogrammus randalli* Fricke, 1983				2016	C	RS	Co	Ss	I
*Dussumieria elopsoides* Bleeker, 1849				1952	**Inv**	RS	Co	P	I,II
*Encrasicholina punctifer* Fowler, 1938				2014	C	RS	Co	P	II
*Epinephelus coioides* (Hamilton, 1822)				2014	C	RS	Co	Hs	I
*Equulites klunzingeri* (Steindachner, 1898)			1966	1942	**Inv**	RS	Co	Ss	I-III
*Equulites popei* (Whitley, 1932)				2014	**Inv**	RS	Co	Ss	II-IV
*Etrumeus golanii* DiBatistta, Randall and Bowen, 2012			2002	1994	**Inv**	RS	Co	P	I,II
*Fistularia commersonii* (Rüppell, 1835)			2002	2001	**Inv**	RS	Co	Hs/Ss	I,II
*Fistularia petimba* Lacepède, 1803				2016	**Inv**	RS	Co	Ss	II
*Hazeus ingressus* Engin, Larson, Irmak, 2018				2015	C	RS	Co	Ss	II
*Hemiramphus far* (Forsskål, 1775)			2009	1942	E	RS	Co	P	I
*Heniochus intermedius* Steindachner, 1893				2002	C	RS	Co	Hs/Ss	I
*Herklotsichthys punctatus* (Rüppell, 1837)				1984	E	RS	Co	P	I,II
*Hippocampus fuscus* Rüppell 1838				2003	E	RS	Co	Ss	I
*Jaydia queketti* (Gilchrist, 1903)			2009	2004	E	RS	Co	Ss	I-III
*Jaydia smithi* Kotthaus, 1970				2008	E	RS	Co	Ss	I,II
*Lagocephalus guentheri* (Richardson, 1844)		2007	1966	1949	**Inv**	RS	Co	Ss	I,II
*Lagocephalus sceleratus* (Gmelin, 1789)	2017	2008	2003	2004	**Inv**	RS	Co	Ss	I,II
*Lagocephalus suezensis* Clark & Gohar, 1953			2001	1998	**Inv**	RS	Co	Ss	I,II
*Liza carinata* (Valenciennes, 1836)				1955	E	RS	Co	P	I
*Lutjanus argentimaculatus* (Forsskål, 1775)			2018		C	RS	Co	?	I
*Monotaxis grandoculis* (Forsskål, 1775)				2007	C	RS	Co	Ss	I,II
*Nemipterus randalli* Russell, 1986			2011	2007	**Inv**	RS	Co	Ss	I,II
*Ostorhinchus fasciatus* (White, 1790)			2011	2009	E	RS	Co	Ss	I,II
*Ostracion cubicus* Linnaeus, 1758				2017	C	RS	Co	Hs	II
*Oxyurichthys petersi* (Klunzinger, 1871)			1991	1991	E	RS	Co	Ss	I-III
*Parablennius thysanius* (Jordan & Seale, 1907)				2013	C	IP	S	Hs	I
*Paranthias furcifer* (Valenciennes, 1828)			2020	2003	C	AT	?	Hs	I,II
*Parexocoetus mento* (Valenciennes, 1846)			1966	1966	E	RS	Co	P	I
*Parupeneus forskalli* (Fourmanoir & Guézé, 1976)			2016	2000	**Inv**	RS	Co	Ss	I,II
*Pelates quadrilineatus* (Bloch, 1790)				1984	E	RS	Co	Ss	I,II
*Pempheris rhomboidea* Kossmann & Räuber, 1877		2020	1994	1983	E	RS	Co	Hs	I,II
*Petroscirtes ancylodon* Rüppell, 1838			2005	1997	E	RS	Co	Hs/Ss	I
*Planiliza haematocheilus* (Temminck & Schlegel, 1845)	1992	1995	1995		**Inv**	PO	Aq	P	I
*Platax teira* (Forsskål, 1775)				2006	C	RS	Co	Hs	I
*Plotosus lineatus* (Thunberg, 1787)				2016	C	RS	Co	Hs/Ss	II
*Pomacanthus imperator* (Bloch, 1787)				2019	C	RS	Co	Hs	I
*Pomadasys stridens* (Forsskål, 1775)			2015	2009	E	RS	Co	Ss	I,II
*Priacanthus hamrur* (Forsskål, 1775)				2017	C	RS	Co	Ss	II
*Priacanthus prolixus* Starnes, 1988				2016	C	RS	Co	Ss	III
*Priacanthus sagittarius* Starnes, 1988				2017	C	RS	Co	Ss	III
*Pteragogus trispilus* Randall, 2013			2002	1998	E	RS	Co	Hs/Ss	I,II
*Pterois miles* (Bennett, 1828)			2015	2014	**Inv**	RS	Co	Hs	I,II
*Rachycentron canadum* (Linnaeus, 1766)			2013		C	CT	Co?	P	II
*Sargocentron rubrum* (Forsskål, 1775)			1949	1949	E	RS	Co	Hs	I-III
*Saurida lessepsianus* (Russell, Golani and Tikochinski, 2015)			1973	1951	**Inv**	RS	Co	Ss	I-III
*Scarus ghobban* Forsskål, 1775				2013	C	RS	Co	Hs	I
*Scomberomorus commerson* Lacepède, 1800			1994	1981	E	RS	Co	P	I,II
*Siganus luridus* (Rüppell, 1829)			1973	1973	**Inv**	RS	Co	Hs	I,II
*Siganus rivulatus* Forsskål, 1775		2019	1966	1942	**Inv**	RS	Co	Hs	I,II
*Sillago suezensis* Golani, Fricke and Tikochinski, 2014			2004	1983	E	RS	Co	Ss	I,II
*Sphyraena chrysotaenia* Klunzinger, 1884			1966	1955	**Inv**	RS	Co	P	I,II
*Sphyraena flavicauda* Rüppell, 1838				2001	**Inv**	RS	Co	P	I,II
*Stephanolepis diaspros* Fraser-Brunner, 1940		2011	1943	1949	E	RS	Co	Hs/Ss	I,II
*Stolephorus insularis* Hardenberg, 1933				2012	C	RS	Co	P	II,III
*Synanceia verrucosa* Bloch & Schneider, 1801				2011	C	RS	Co	Ss	II
*Synchiropus sechellensis* Regan, 1908				2014	E	RS	Co	Ss	II,III
*Torquigener flavimaculosus* Hardy & Randall, 1983			2014	2002	**Inv**	RS	Co	Ss	I,II
*Trachurus indicus* Nekrasov, 1966				2004	C	RS	Co	P	I-III
*Trypauchen vagina* (Bloch & Schneider, 1801)				2010	E	RS	Co	Ss	II
*Tylerius spinosissimus* (Regan, 1908)				2010	C	RS	Co	Ss	II,III
*Upeneus moluccensis* (Bleeker, 1855)			1973	1942	**Inv**	RS	Co	Ss	I-IV
*Upeneus pori* Ben-Tuvia & Golani, 1989			2000	1942	**Inv**	RS	Co	Ss	I,II
*Vanderhorstia mertensi* Klausewitz, 1974			2010	2008	E	RS	Co	Ss	I-III
**MAMMALIA**									
*Sousa plumbea* (G. Cuvier, 1829)				2016	C	RS	Co	P	I

The habitat and depth preferences of aliens along the coasts together with their possible origins and establishment success are also given. BS: Black Sea, SM: Sea of Marmara, AS: Aegean Sea, LS: Levantine Sea, ES: Establishment Success (E: Established, C: Casual,Inv: Invasive), O: Origin (IP: Indo-Pacific, RS: Red Sea, AT: Atlantic, NA: North Atlantic, WA: Western Atlantic, ST: Subtropical Atlantic/Pacific, IO: Indian Ocean, PG: Persian Gulf, PO: Pacific Ocean, TA: Tropical Atlantic, CT: Circumtropical,Unk: Unknown) PW = Pathway (Co: Corridor/Canal (vector: Suez Canal), S: Ships, Aq: Aquaculture activities), H: Habitat [Hs: Hard Substratum (including epibiontic species on algae, sponges), Ss: Soft Substratum (including soft substrata with phanerogames), P: pelagic, Pz: parasite], DR: Depth Range (I: 0–10 m, II: 11–50m, III: 51–100 m, IV: 101–200 m, V: 201–400, VI: 401–500 m, VII: >500m).

**Fig 2:**
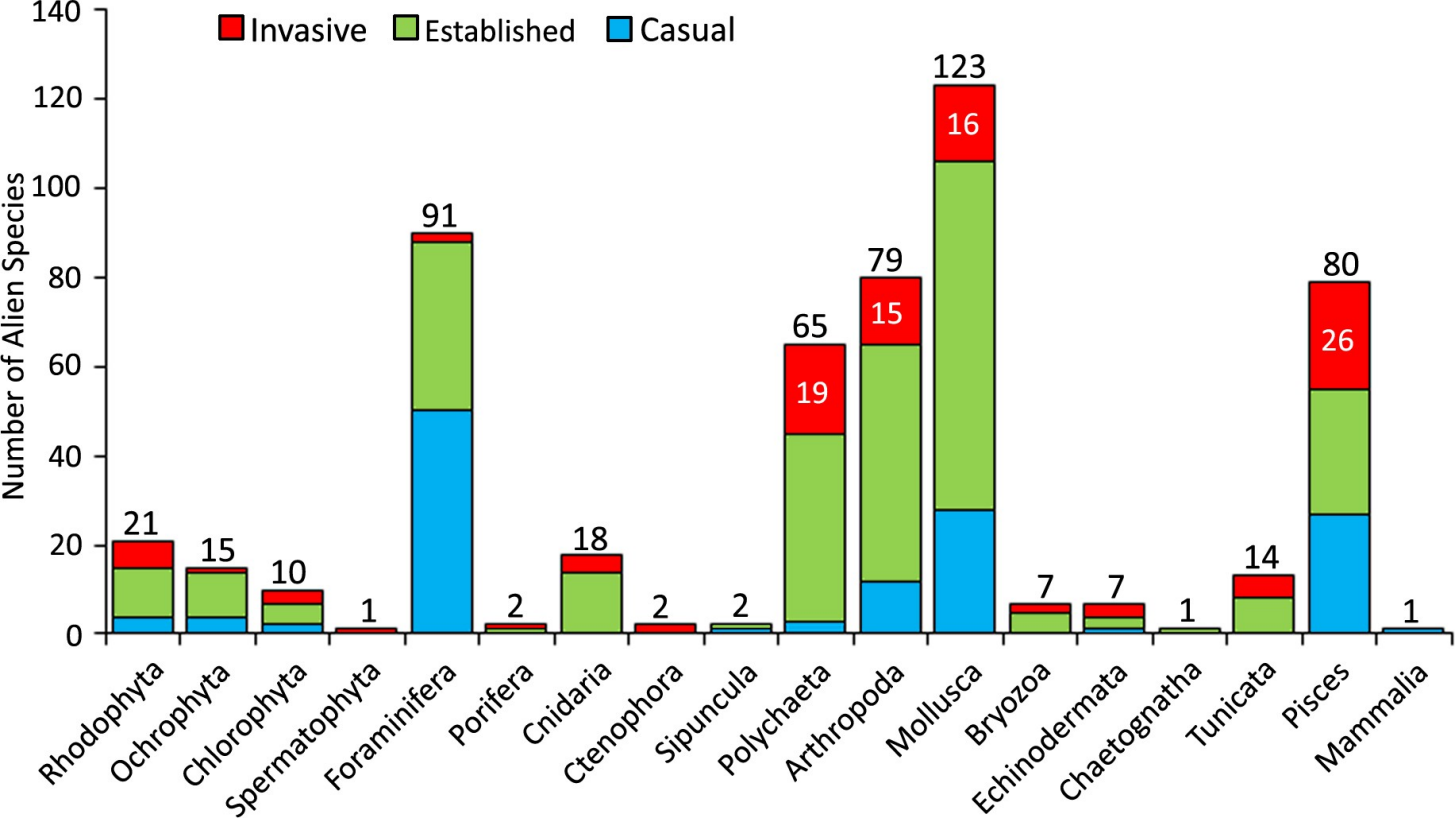
Number of invasive, established and casual alien species in each taxonomic group.

Among the established species, 105 species have invasive characters at least in one zoogeographic region, comprising 19% of all alien species and 26% of established alien species. Mollusca ranked first in terms of the number of alien species (123 species), followed by Foraminifera, Arthropoda and Pisces (Fig 2). The groups with the highest casual alien species are Foraminifera (51 species), Mollusca (27 species) and Pisces (28 species). Casual species are absent in 7 taxonomic groups. Established species accounted for more than 60% of total number of species in 5 groups [Chaetognatha (100%), Cnidaria (78%), Bryozoa (71%), Ochrophyta (67%) and Arthropoda (66%)]. The number of invasive alien species varied among groups, with the highest scores being calculated in Pisces (26 species) and Polychaeta (19 species). All species in Ctenophora (*Beroe ovata* and *Mnemiopsis leidyi*) and Spermatophyta (*Halophila stipulacea*) are invasive. The other groups with the highest percentage of invasive alien species are Echinodermata (57%), Porifera (50%), Tunicata (36%), Pisces (33%) and Chlorophyta (30%).

**Fig 3:**
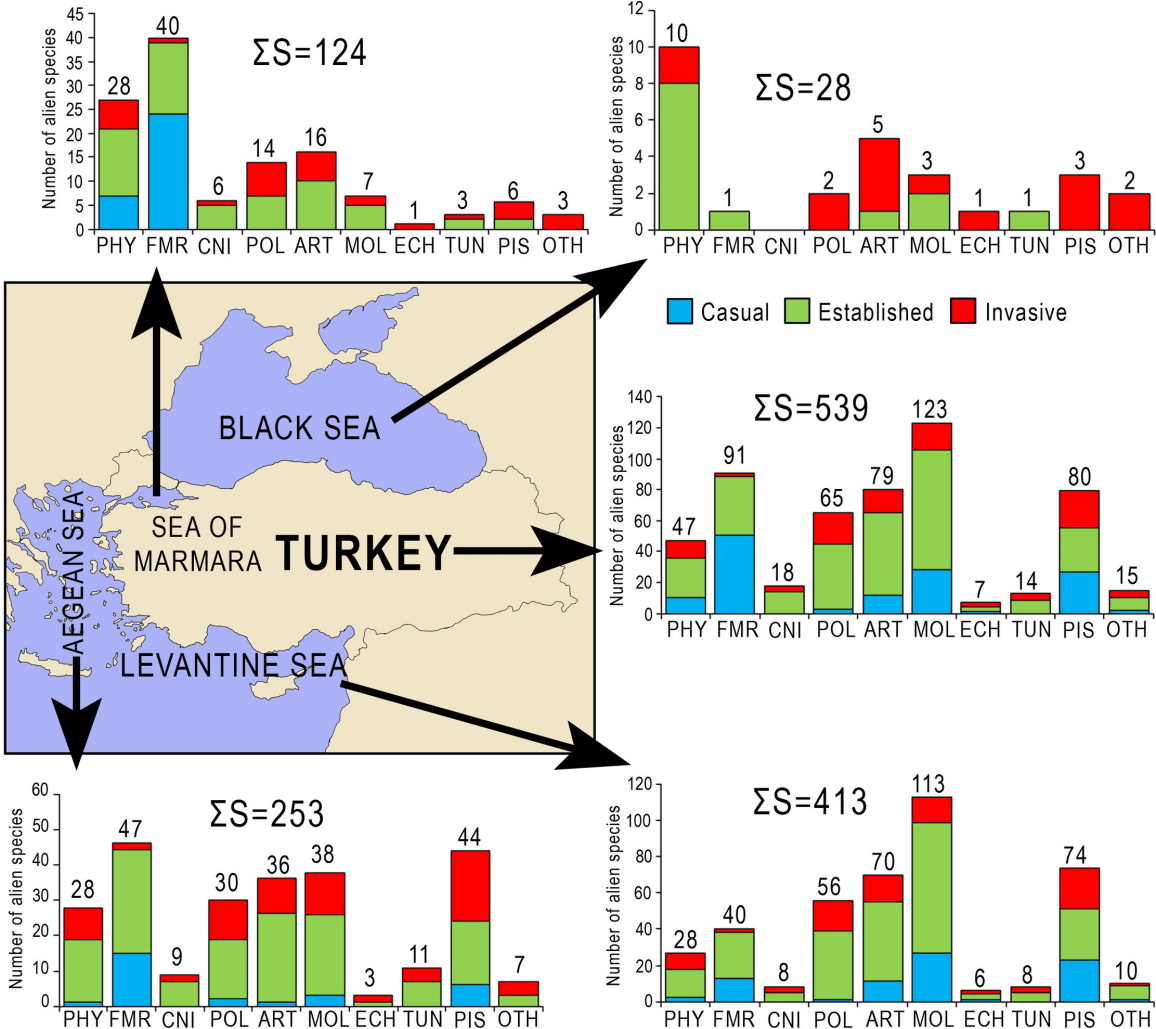
Number of alien species in four seas surrounding Turkey, and their distributions to major groups and their establishment success in each sea. PHY: Phytobenthos, FMR: Foraminifera, CNI: Cnidaria, POL: Polychaeta, ART: Arthropoda, MOL: Mollusca, ECH: Echinodermata, TUN: Tunicata, PIS: Pisces, OTH: Others.

The number of alien species found in seas surrounding Turkey ranged from 28 (Black Sea) to 413 (Levantine Sea) (Fig 3). The decrease in the number follows a clockwise direction along the coasts, from south to north, from proximity to farness with regards to the Suez Canal, which is the main vector of the species introduction in the Mediterranean Sea [3, 7, 26]. The percentage of invasive alien species within the total number of alien species differs among the regions, with the highest percentage being estimated in the Black Sea. Having brackish water body and high primary productivity makes this sea vulnerable to biotic invasions [33]. The excessive proliferation of invasive species transported by ships in the region has had enormous ecological and economic consequences [7]. The percentage of invasive fish species in the total number is generally high in seas, but the decrease in the percentage follows an anti-clockwise direction from the Black Sea (100%) to the Levantine Sea (30%), that is also apparent for other groups such as Polychaeta and Arthropoda.

**Fig 4:**
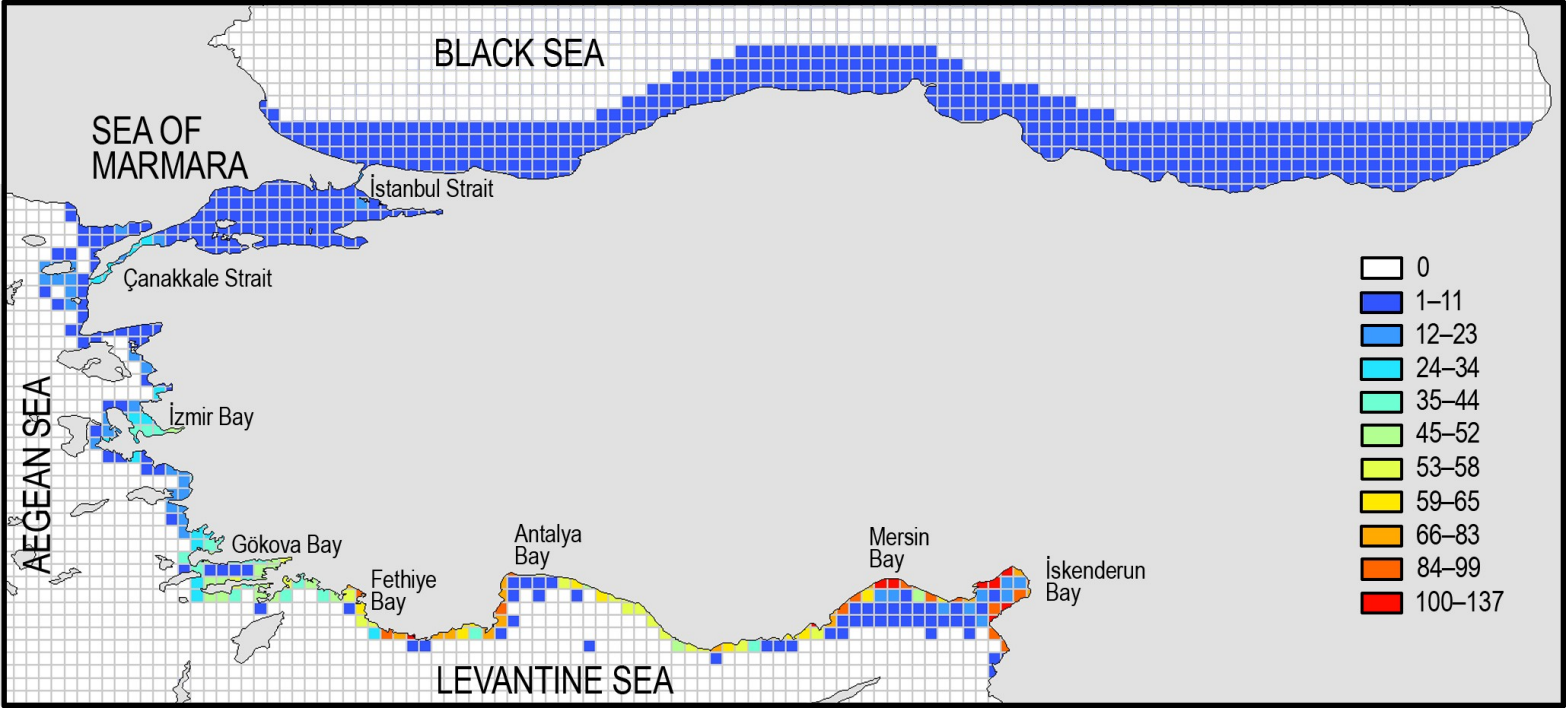
The number of alien species found in grids of 15x15 km along the coasts of Turkey.

The coasts of Turkey have been densely invaded by alien species, there is no grid area (15x15 km) along the coast remained unoccupied by alien species. The worst conditions are prevailing on the Levantine coast, where some grids in İskenderun, Mersin and Antalya Bay have more than 100 alien species. The south Aegean Sea is also represented by higher number of alien species, and the number diminishes when moving to north, except for the Çanakkale Strait, where relatively higher number of alien species has been reported. According to the grid map depicted in Fig 4, six hot-spot areas can be identified for the settlement of alien species, namely İskenderun Bay, Mersin Bay, Antalya Bay, Fethiye Bay, Gökova Bay and İzmir Bay, where more than one pathway of species introductions (e.g., corridor and ship) are present.

**Fig 5:**
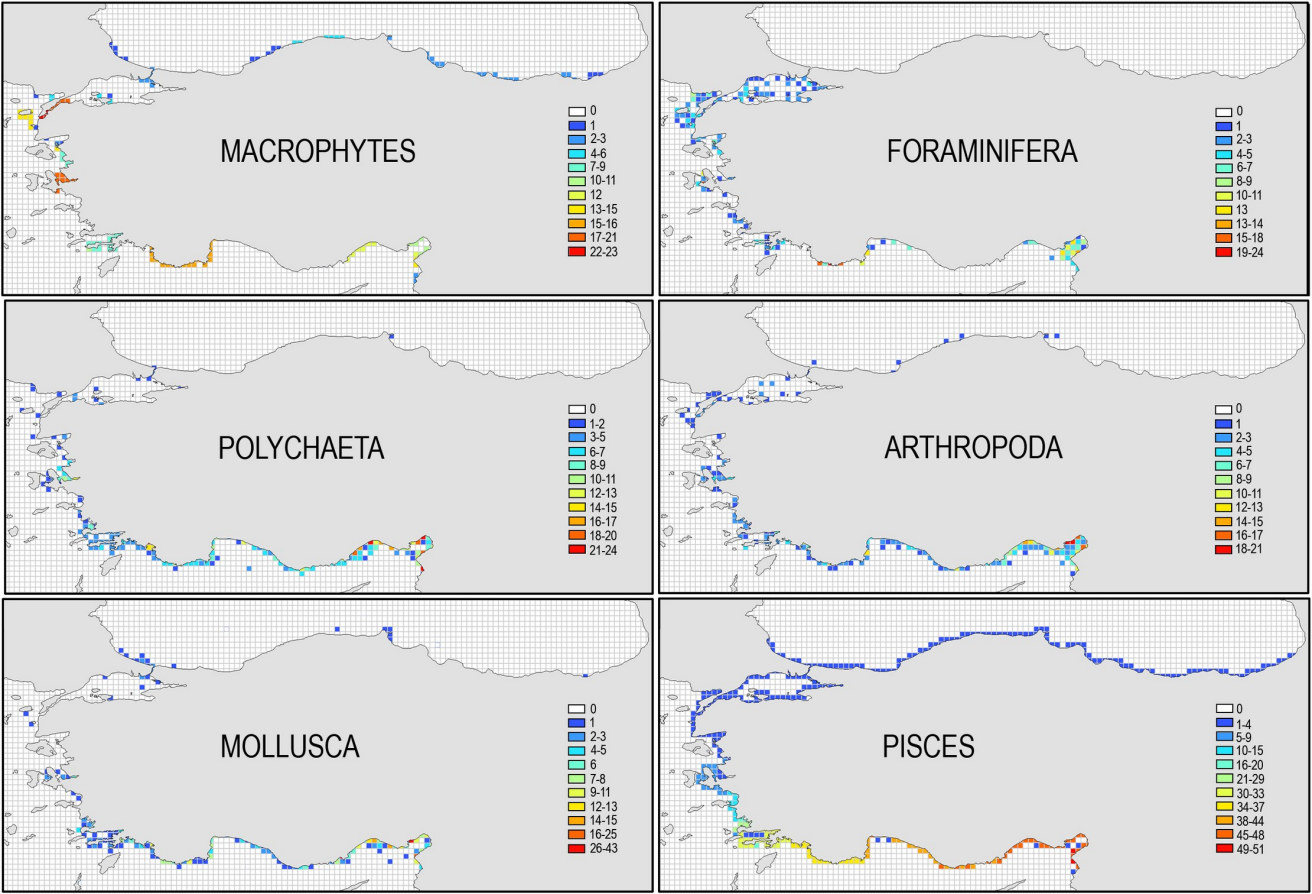
The number of alien species belonging to major groups found in grids of 15x15 km along the coasts of Turkey.

The distribution pattern of alien species found in the general picture depicted in Fig 4 is also applicable for the distribution of alien species belonging to major taxonomic groups (Fig 5). The highest scores in grids were estimated for Pisces (maximum 51 species in İskenderun Bay), followed by Mollusca (43 species). The maximum number of alien species in grids (21–24 species) is more or less similar for other major groups. İskenderun Bay hosts the highest number of alien species for all major groups, except for the group Macrophytes, which has three hot-spots areas, distant from each other, namely Çanakkale Strait, İzmir Bay and Antalya. This is not related to the hydrographic characters of the areas, but primarily related to the availability of taxonomic experts in the institutions located in the areas which have been studied and monitored comprehensively over time. Such biodiversity pattern was also previously noted along the coasts of Turkey for many taxonomic groups (see [25]).

**Fig 6:**
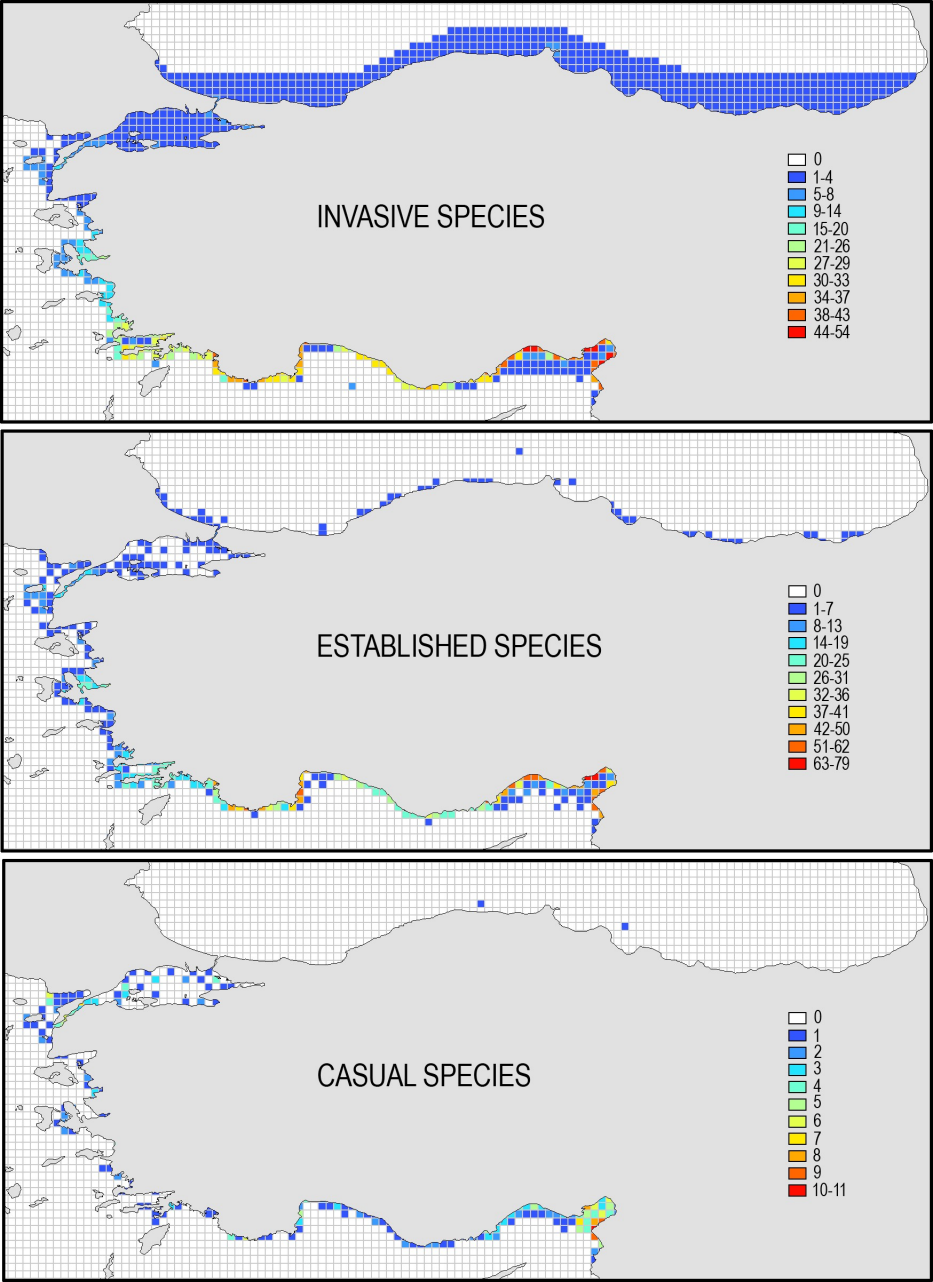
The distribution of invasive, established and casual alien species along the coasts of Turkey.

Invasive and established alien species have been reported on all grids along the coasts, but the casual species are general found on the Levantine coast, especially in Iskenderun Bay, which acts as a gate to the marine waters of Turkey for the introduced Red Sea species (Fig 6). Some grids in İskenderun and Mersin Bays had the number of invasive and established alien species higher than 40. The majority of them are the Red Sea invaders that have been colonizing all shallow-water benthic pelagic habitats including harbors [34] and estuarine areas [35].

The species list presented here complies with the previous lists prepared for countries or specific taxonomic groups in the Mediterranean Sea. However, there are some discrepancies among the lists, mainly due to difference in the evaluations of available data by experts. For example, the presence of *Eurythoe complanata*, *Neopseudocapitella brasiliensis*, *Metasychis gotoi* and *Pista unibranchia* in the Mediterranean were reported as questionable [36], whereas we considered them as established alien species. The judgements by [36] were not based on new molecular and morphological data, or comparisons of the Mediterranean specimens with the type specimens of the species. The presence of *E*. *complanata* was also previously regarded as questionable because of the confusion with the native, but very poorly described species *E*. *syriaca* [7, 37]. However, [38] examined some *Eurythoe* specimens collected from different parts of the Mediterranean and identified them as *E*. *complanata*. The presence of *M*. *gotoi* and *Pista unibranchia* in the Red Sea was confirmed by [39] and [40]. In addition, the morphological features of *N*. *brasiliensis*, *M*. *gotoi* and *P*. *unibranchiata* match with their original descriptions (MEÇ, personal data), so they likely occur in the region. However, further studies (especially comparison with type specimens) are needed to clarify the real taxonomic identities of the specimens reported under these names from the Mediterranean Sea.

In a new revision on alien foraminiferans [41], 44 established alien species were reported from the Mediterranean Sea, almost half the species presented in our list. One of the main reasons of this great difference in the number of species is that the authors have not considered many of the species recorded as alien from the Mediterranean coasts of Turkey and the Sea of Marmara as well as the recently recorded alien species from the Mediterranean coasts of Turkey. Besides, the authors have excluded the species which have fossil records in the Eastern Mediterranean. *Sorites orbiculus*, which is common in the eastern Mediterranean, was also found in cores from Ashqelon (Israel), dated back to 320–2 ka BP [42–44]. However, the genetic analysis showed that the population found in Shikmona (Israel) was genetically identical to a population of it living in the Gulf of Elat (Red Sea) [45]. One of the most invasive alien foraminifer species, *Amphistegina lobifera*, was found in the core materials from Mersin (Turkey), dated back to 227.3 ± 17.8, ka BP [46]. Some Indo-Pacific foraminifer species were also found together with the Mediterranean species in the Quaternary sediments collected from the Asi River (Orontes) Delta [47]. It was suggested that these species might have been introduced to the Eastern Mediterranean much before the opening of the Suez Canal. However, rDNA analyses on *A*.*lobifera* showed that both the Mediterranean and the Red Sea populations are genetically clustered together and distinct from the Australian populations [48], suggesting that the population of *A*.*lobifera* currently spreading in the Mediterranean is originated from the Red Sea.

### What changed after the last update?

**Fig 7:**
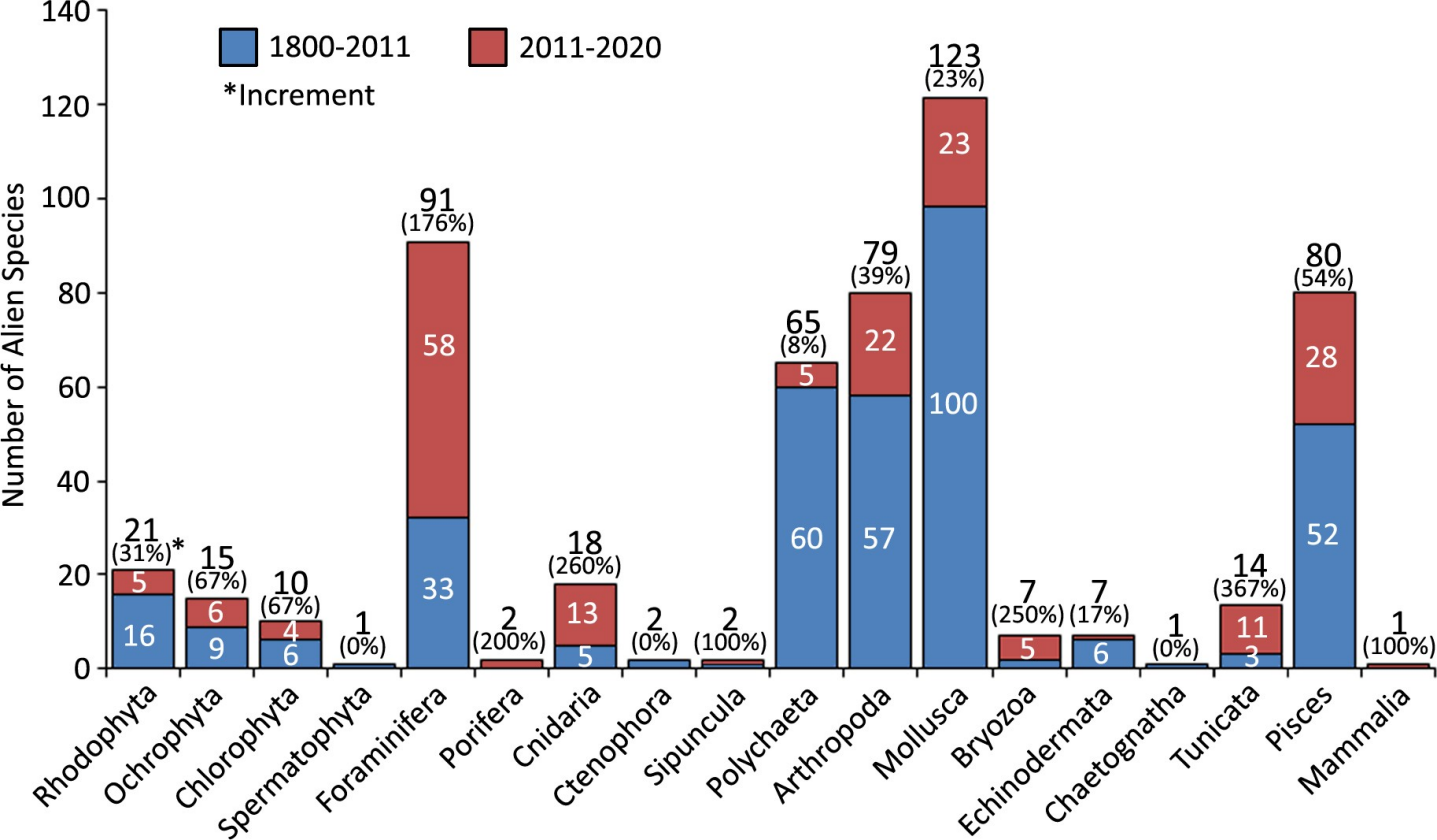
Changes in the number of alien species of taxonomic groups after the 2011’s species list.

After the last update (2011 update, [7]), a total of 184 new alien species have been registered in the regions, bringing the number of alien species from 359 to 539. The major increments are seen in Tunicata (367%), Cnidaria (260%), Bryozoa (250%) and Porifera (200%) (Fig 7). Based on new evidence (see [4]), the present study classified some ascidians (*Styela plicata*, *Ascidiella aspersa* and *Diplosoma listerianum*) as aliens, which were known as native from the region for a long time [49–51]. Another high increment (176%) was encountered in Foraminifera. This is partly due to the new species additions (58 species) and party due to the inclusions of some species that were not treated as aliens in the 2011’s species list, because of the lack of enough data for the assessment of their status as native or alien.

The establishment success or status of alien species has been changed for several species since 2011. For example, the algal species such as *Acanthophora nayadiformis*, *Ganonema farinosum*, *Cladosiphon zosterae* and *Pylaiella littoralis*, which were previously classified as questionable or cryptogenic are considered herein as established alien species, because of the new evidences on the proper taxonomic entities of these species [52, 53] and their pathways of introductions: *A*. *nayadiformis* and *G*. *farinosum* might have been introduced by multiple vectors (shipping or via the Suez Canal), while others (*C*. *zosterae* and *P*. *littoralis*) by shellfish and oysters farming [54]. Based on the new data accumulated since 2011, ten casual species (*Phyllorhiza punctata*, *Pisione guanche*, *Monocorophium sextonae*, *Eurycarcinus integrifrons*, *Penaeus aztecus*, *Pilumnus minutus*, *Sticteulima lentiginosa*, *Monotigma fulva*, *Odostomia lorioli* and *Pomadasys stridens*) in the 2011’s species list have turned to become established alien species. In the last decade, a radical change observed in the establishment success of *Parupeneus forskalli*, which was very rare (casual) before 2011, is now an invasive alien species in the region, having expanded its distribution range to the Aegean Sea [55].

### Yearly changes in the number of alien species

**Fig 8:**
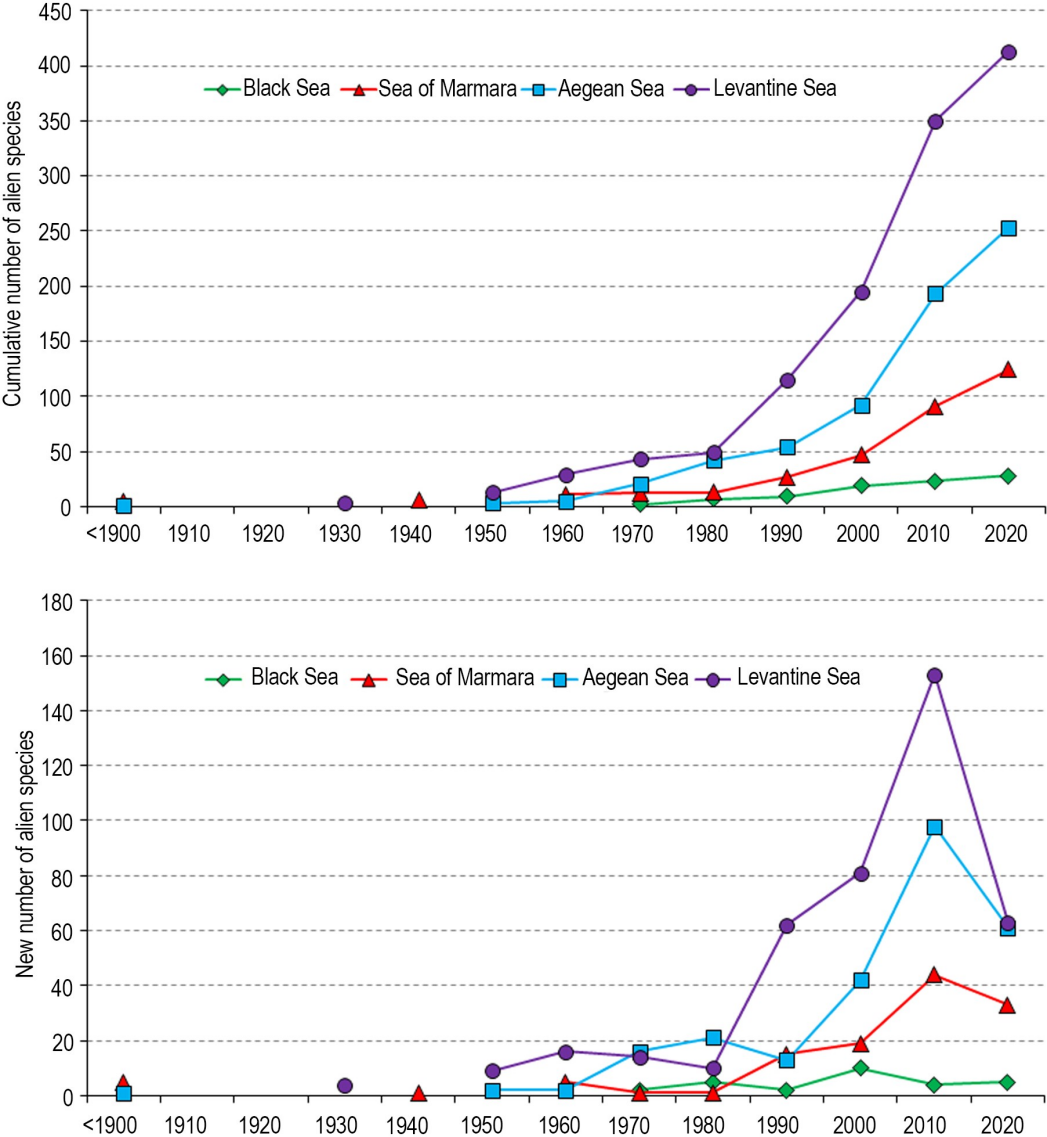
Cumulative (upper) and new (lower) number of alien species in seas surrounding Turkey over years.

The cumulative number of alien species increases over years in all seas surrounding Turkey (Fig 8). The new alien species are being reported from all coasts, but especially from the east Levantine Sea, because of the impact of the Suez Canal as a primary vector. However, as shown in the last time interval (2010–2020), the trend slows down over time. The number of new alien occurrence (collection date) decreased in the last 10 years (2010–2020) on the coasts of Turkey, when compared to the previous time intervals since 1980. The sharp decrease was encountered in the Levantine Sea, where 153 new alien occurrences were reported between 2000 and 2010, whereas 63 species (a 59% decrease) were determined between 2010 and 2020 (Fig 8). Such decreases were also estimated between the periods in the Aegean (-38%) and the Sea of Marmara (-25%), but on the contrary an increase (25%) was observed in the Black Sea. The increase and decrease in the numbers are partly related to the scientific efforts devoted to assessing of alien species in the areas, but also partly related to the number of new incomers from different pathways and range extensions of the alien species. For example, it is very surprising to see that the Black Sea’s brackish water habitats started to host the Red Sea originated alien species, such as the puffer fish *Lagocephalus sceleratus* [56] and the shrimp scad *Alepes djedaba* [57]. As in the case of the Levantine Sea, whether the pufferfish would be a nuisance for the Black Sea or not is currently a mystery.

### Primary pathways of species introductions

The assessment of primary pathways of species introduction sometimes remains very controversial, as more than one pathway can be incorporated with the species introduction. The step-by-step distributional patterns of the Red Sea species, so called Lessepsian species, can indicate an introduction via the corridor, the Suez Canal. It is sometimes hard to assess the pathway or even the assignation of species as native or alien for suddenly appearing species with high abundances in one area far from the Canal. For example, *Syllis ergeni*, which was originally described in Izmir Bay (Aegean Sea) in 2005 [58], was previously considered as an endemic species for the Mediterranean Sea, but due to its high abundance in the shallow-water benthic habitats in the Levantine Sea and the presence of it in the Red Sea [59], it was classified as alien species.

**Fig 9:**
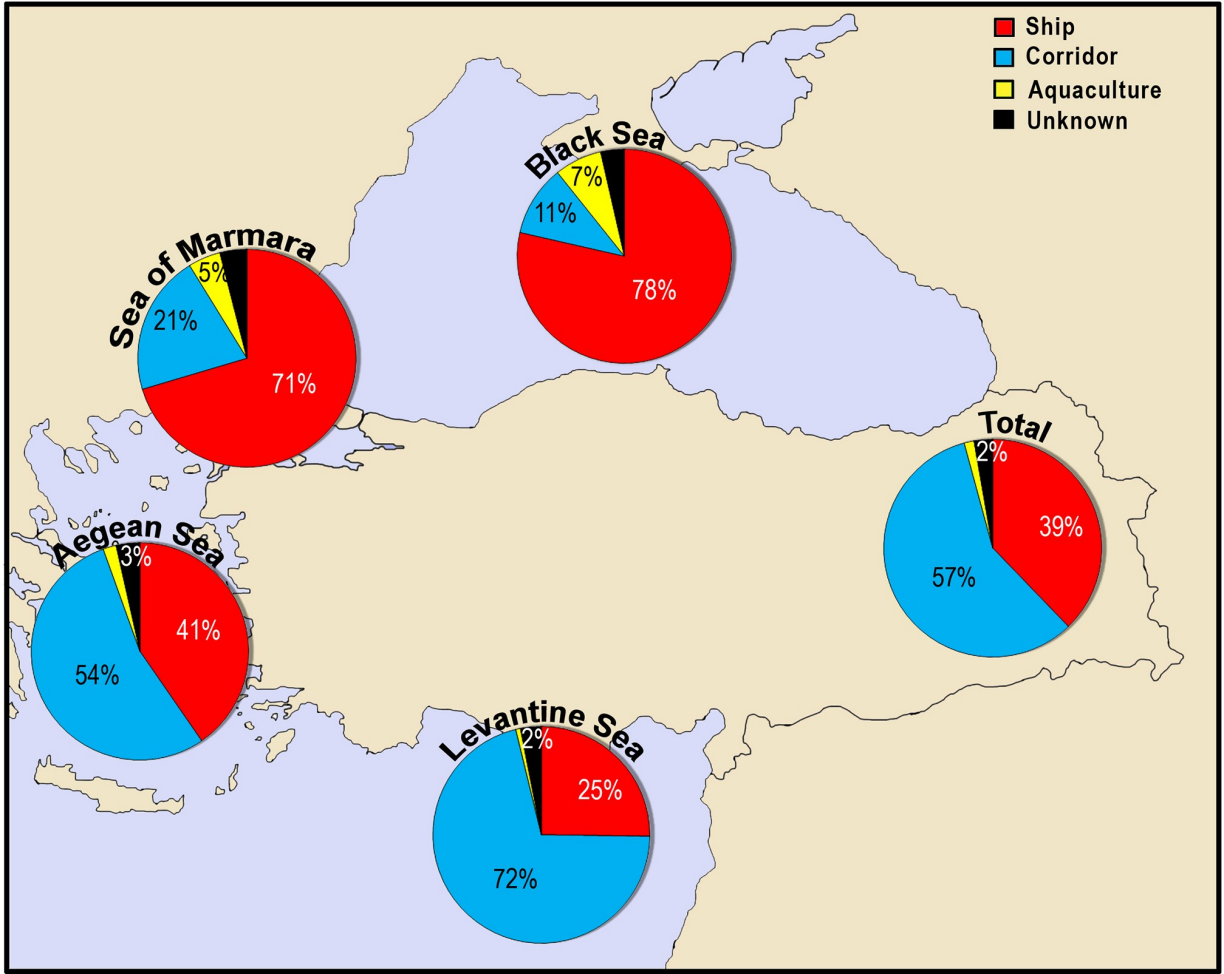
The primary pathways of introduction for the alien species along the coasts of Turkey.

In this review, we evaluated the primary pathways of alien species as almost all species of the introduced Red Sea species have reached to the coasts of Turkey by natural dispersal mechanism from the neighboring countries, following the main current direction prevailing in the region. There are few numbers of ship-transferred species (e.g., *Asterias rubens* and *Streblospio gynobranchiata*) that have been firstly reported from the coasts of Turkey within the Mediterranean Sea. The importance of the Suez Canal in the species introduction diminishes when moving from south to north in Turkey, accounting for 72% of species introductions in the Levantine Sea, whereas it comprised only 11% of species introductions in the Black Sea, where majority of the introductions (78%) were carried out by ships (ballast water or hull fouling) (Fig 9). Eight established alien species, namely the brown algae *Scytosiphon dotyi* and *Cutleria multifida*, the green alga *Ulva australis*, the shrimp *Penaeus merguiensis*, the gastropod *Crepidula fornicata*, the bivalves *Magallana gigas* and *Ruditapes philippinarum* and the fish *Planiliza haematocheilus*, were introduced to the Mediterranean/Black Sea via aquaculture activities and then secondarily to the coasts of Turkey.

**Fig 10:**
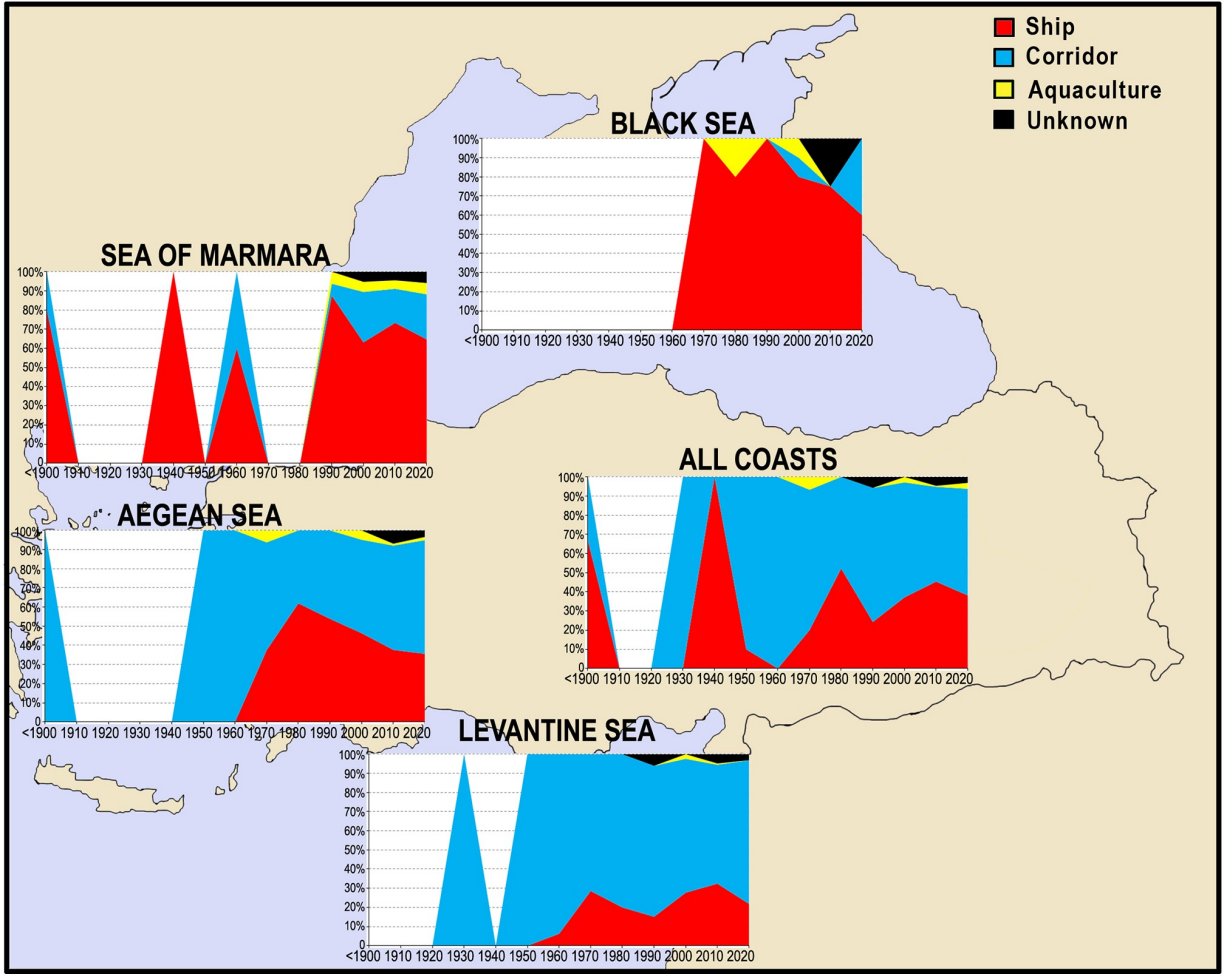
The trends in the importance of primary pathways in species introductions in seas per decade.

The trends in the importance of pathways in species introductions in all seas over years are indicated in Fig 10. Since 1990, the number of corridor-borne species increased in the Aegean Sea (110 species entered) and the Sea of Marmara (21 species entered), as a consequence of the range expansions of the Red Sea originated species from the Levantine Sea. In the last decade, the number of corridor-borne species entering the Black Sea has increased approximately two times when compared to other years, indicating that the sea is also under threat by the Red Sea species of wider ecological valances such as *Lagocephalus sceleratus*. In the last decade, the corridor-borne species comprised higher percentages in species introductions in all seas. An increasing trend in the number of ship-borne species is evident between the periods 1990–2010 in the Levantine Sea, but its importance in the species introduction was dropped from 32% to 22% in the last decade. Considering data from all coasts, there are four clear peaks in the number of ship-borne species over years: 65% (4 species) in <1900, 100% (1 species) in 1931–1940, 52% (12 species) in 1971–1980 and 45% (88 species) in 2001–2010.

### Origins of alien species

**Fig 11:**
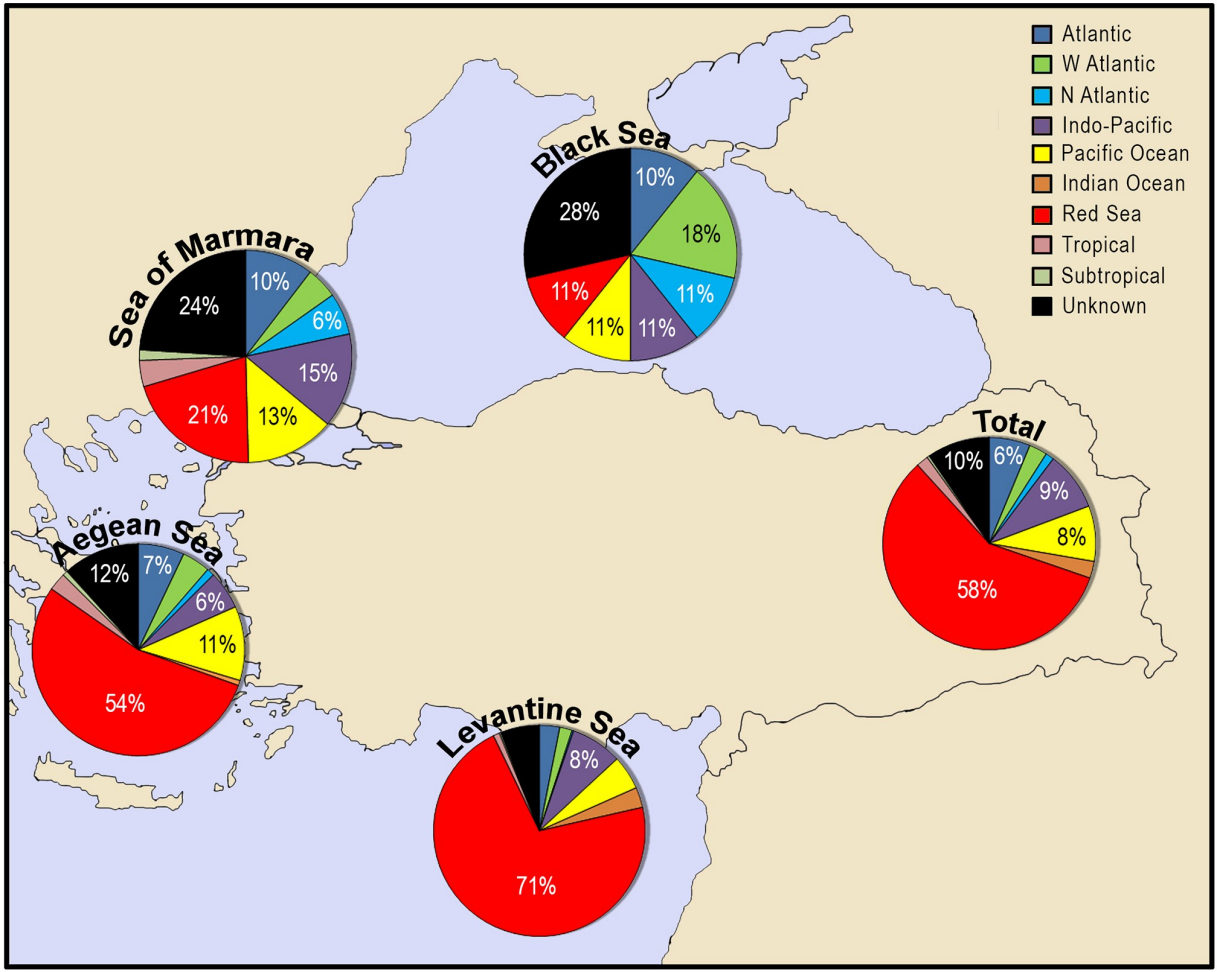
The origins of alien species reported along the coasts of Turkey.

Most alien species reported from the coasts of Turkey were originated from the Red Sea (58%), due to the proximity of the country to the Suez Canal (Fig 11). The shipping activities transported 39% of total number of alien species, mainly from the Indo-Pacific area (20%) and the Atlantic Ocean (10%). However, the origins of alien species differ from the seas surrounding Turkey. Majority of the species in the Black Sea was originated from the western Atlantic (25%), whereas those of the Sea of Marmara (21%), Aegean Sea (54%) and Levantine Sea (72%) were originated from the Red Sea. The north Atlantic-originated species such as *Adelosina colomii*, *Diadumene cincta*, *Beroe ovata* and *Asterias rubens* occurred solely in the Sea of Marmara and Black Sea, at least at the time being.

### Habitat and depth preferences

**Fig 12:**
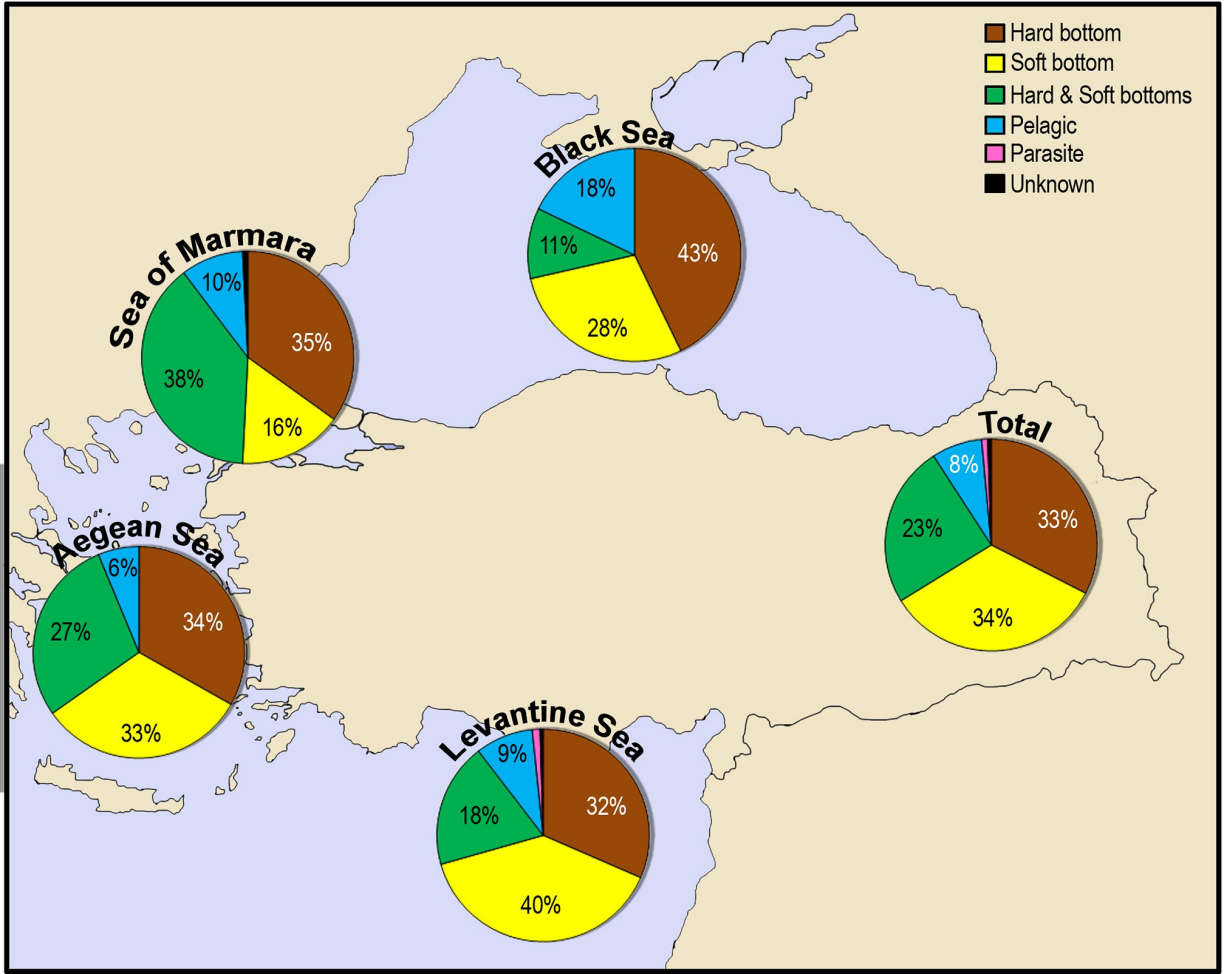
The habitats preferences of alien species on the coasts of Turkey.

The habitat preferences of alien species varied along the coasts of Turkey (Fig 12). In general, majority of alien species were recorded from the soft bottom (309 species), accounting for 57% of total number of species. The pelagic realm hosted 42 alien species (8%). One rhizocephalan and 4 copepod parasitic species were found on the alien and native species only in the Levantine Sea: *Heterosaccus dollfusi* on the alien crab *Charybdis longicollis* [60]; *Caligus lagocephali* (cited as *C*. *fugu*) on the pufferfish, *Lagocephalus suezensis* and *L*. *guentheri* [61]; *Mitrapus oblongus* on the native fish *Sardinella aurita* [62]; *Lernanthropus callionymicola* on the alien fish *Callionymus filamentosus* [63]; and *Taeniacanthus lagocephali* on the alien fish *Lagocephalus guentheri* [61]. The habitat preferences of alien species are almost similar each other in the Sea of Marmara, Aegean Sea and Levantine Sea, but hard-bottom species are numerically dominant in the Sea of Marmara (73%) and Aegean Sea (61%), whereas soft-bottom species in the Levantine Sea (58%). The Black Sea’s alien species mainly occupied hard bottom (totally 54%). The percentage of pelagic alien species (18%) in the Black Sea is at least two times higher than those estimated in other seas.

**Fig 13:**
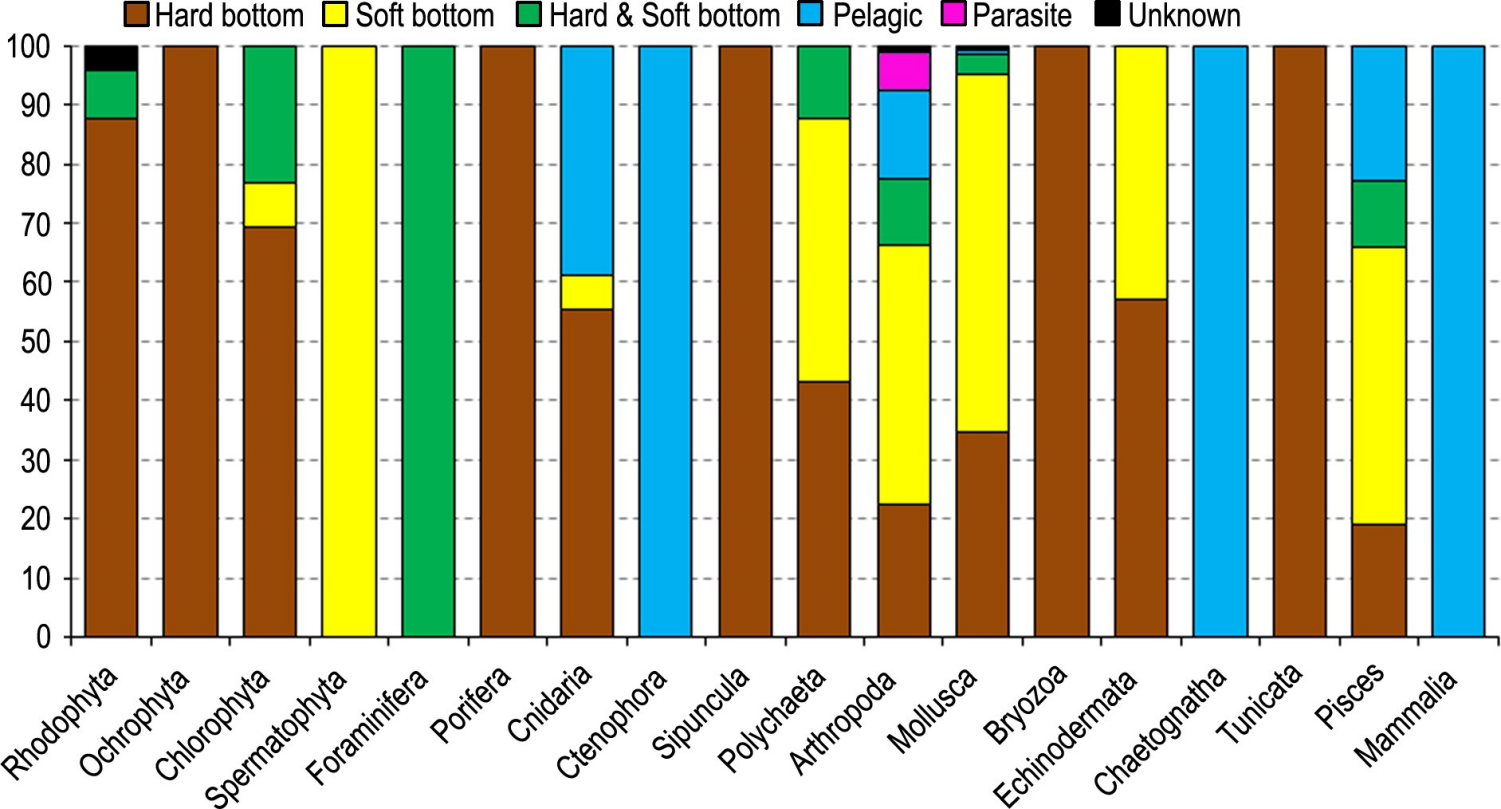
Habitat preferences of alien species in taxonomic groups reported from the coasts of Turkey.

A total of 5 taxonomic groups (Ochrophyta, Porifera, Sipuncula, Bryozoa and Tunicata) occurred only on hard substrata, whereas the phanerogame *Halophila stipulacea* and majority of the species in Polychaeta, Arhtropoda, Mollusca and Pisces occupied soft substrata (Fig 13). All species in Scyphozoa, Ctenophora and Mammalia were reported in the pelagic environment. All foraminiferans were found both in hard and soft substrata.

**Fig 14:**
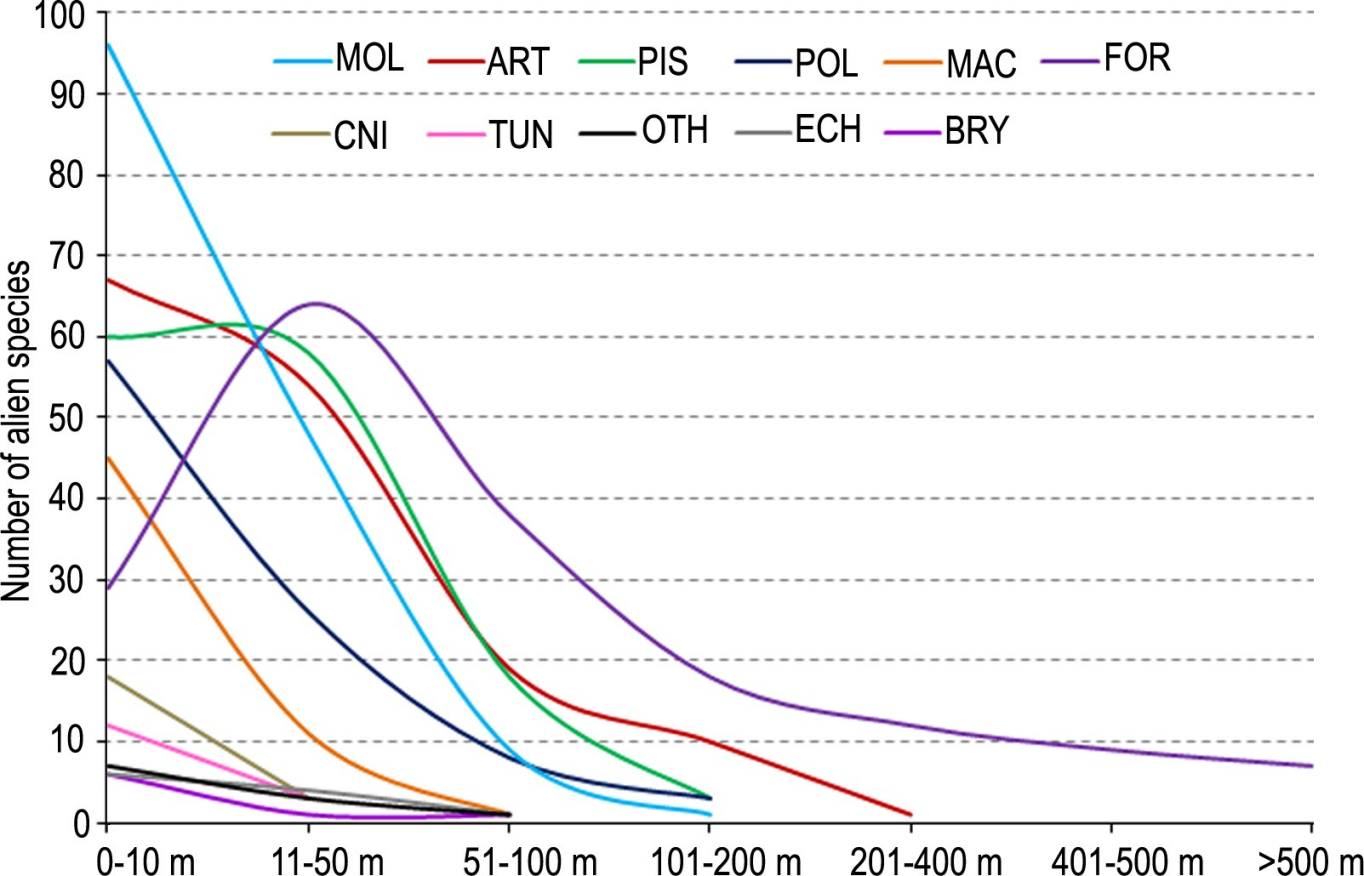
The number of alien species in major taxonomic groups found in the depth intervals.

In all taxonomic groups, large portion of the species (>70% of total number of species) were found in the depth interval 0–10 m, except for Foraminifera that were represented by higher number of species (62%) in the depth interval 11–50 m (Fig 14). All alien species of Cnidaria and Tunicata were found in 0–10 m depth, but a few in 10–50 m. The macrophytes (94%) and Polychaeta (88%) primarily occurred in the shallow water (0–10 m). In general, the number of alien species dropped at least two times at 10–50 m depth, with the exceptions of Foraminifera (increase, +>100%), Pisces (slight decrease -4%) and Echinodermata (-34%). A sharp decrease was seen in the depth intervals 50–100 m (totally 94 species, 17% of total alien species) and 100–200 m (totally 33 species, 6%). Only five groups [Mollusca (1 species), Arthropoda (10 species), Pisces (3 species), Polychaeta (3 species) and Foraminifera (16 species)] occurred at 100–200 m depths. The deep-water (>200 m depth) alien species registered to Arthropoda (1 species, *Trachysalambria palaestinensis*) and Foraminifera (12 species). Among the foraminiferans, *Ammodiscus gullmarensis* (11–500 m), *Astacolus insolitus* (11–500 m), *Melonis affinis* (11–500 m), *Spiroloculina subcommunis* (61–1042 m), *Veleroninoides scitulus* (70–500 m), *Recurvoidella bradyi* (90–500 m) and *Procerolagena gracilis* (104–807 m) are characterized by having a vast depth range, whereas *Pseudoclavulina humilis* (1224 m), *Stainforthia fusiformis* (427–1100 m) and *Candeina nitida* (760 m) only occur in deep waters.

### Questionable species

The muzzled blenny *Omobranchus punctatus*, which was first reported from an aquaculture cage in the port of Ashdod (Israel) in 2003 [64], was reported from the Great Harbour of Antalya (Levantine Sea) in 2013 without any figure and description [65]. Until new evidence is provided, it is better to keep the presence of the species in the region as questionable. A recent checklist of Sea of Marmara fishes [66] included 42 erroneous and/or misidentified species, including several incorrect data for alien taxa (i.e *Atherinomorus forskalii*, *Epinephelus fasciatus*, *Equulites klunzingeri*, *Hemiramphus marginatus* and *Parexocoetus mento*) [67], therefore, records of *Alepes djedaba*, *Sargocentron rubrum* and *Upeneus moluccensis* [68–70] will also be treated as questionable, unless their occurrence in the region is substantiated by further research.

Three bivalve species are treated here as questionable: *Spondylus multisetosus*, which was solely reported from the Levantine Sea by [71], might have been confused with its congeneric *S*. *spinosus* [72]; *Nudiscintilla cf*. *glabra*, which was reported only from the Levantine Sea by [73], has uncertainty in its taxonomic identity; *Antigona lamellaris*, whose presence in the Mediterranean Sea was based on a single shell [74].

### Excluded species

In the present study, most alien species previously classified as questionable in the 2011’s review by [7] have been excluded from the marine alien species inventory of Turkey, because their presence has not been subsequently confirmed from the region and do not have proper descriptions and figures in the relevant papers. In addition, some species were also excluded from the alien list of Turkey under the light of new molecular and morphological findings during the decade.

Since the presence of two algal species, *Gracilaria arcuata* and *Litosiphon laminariae* in the Sea of Marmara, which were found in 1984 [75, 76] have not been re-confirmed by subsequent papers in any place of the Mediterranean Sea, they were considered as misidentification or unsuccessful introductions, so here excluded from the alien species list of Turkey.

Three alien foraminiferan species, namely *Edentostomina culturata*, *Spiroloculina antillarum* and *Elphidium charlottense*, were excluded from the previous list, as they were proved to have fossil records in the region [41, 77, 78].

The colonial scleractinid *Oculina patagonica*, which was found as a patch (ca. 1.5 m in diameter) in only one locality (Akkuyu, Levantine Sea) in 2005 in Turkey [15], has been recently proved to be a native species, based on a molecular study by [18] indicating that the Mediterranean population of it have been long isolated from the western Atlantic and recent outburst of it in the region relates to ongoing environmental changes.

The casual and established polychaete species in the 2011’s review, *Marphysa disjuncta*, *Onuphis eremita oculata* and *Streblosoma comatus*, have been re-identified as *M*. *cinari* [79], *Onuphis eremita* by [80] and *S*. *pseudocomatus* by [81], all being considered as native species. The molecular analysis indicated that the so-called invasive alien species in the Mediterranean, *Hydroides dianthus*, turned to be a native species, but the Black Sea populations of it were introduced from the Gulf of Mexico (west Atlantic) [82].

Although they are absent in the 2011’s species list, [83] classified three copepod crustacean species, namely *Pontellina plumata*, *Pseudocalanus elongatus* and *Pteriacartia josephinae*, as alien species, based on the records from the paper by [84]. However, their alien status was later questioned, and all have been re-evaluated as Atlanto-Mediterranean species [85]. A total of six copepod species (*Acrocalanus longicornis*, *Acrocalanus monachus*, *Calanopia americana*, *Calanopia biloba*, *Calanopia minor* and *Parvocalanus latus*), which were only reported from the Sea of Marmara and Levantine Sea [86, 87] and included in the alien species lists [7, 83], were excluded from the alien species lists due to the lack of proper descriptions of the species in the relevant papers and the lack of confirmation upon their presence in the regions by other scientists.

Two alien crustaceans (*Penaeus semisulcatus* and *Portunus segnis*) included to the faunal list of Sea of Marmara [88] are most probably erroneous identifications of *P*.*kerathurus* and *Callinectes sapidus*, respectively. Likewise, the Black Sea record of *P*.*japonicus* [89] is also a misidentification of the native *P*.*kerathurus*.

The Red Sea invader *Diadema setosum* was reported [90] to have an already established population in the upper layer of the Sea of Marmara (4–6 m depths, collection date: July 2018), which is formed by the Black Sea brackish waters. This new report of this species represents its northernmost distributional point in the Mediterranean, almost 300 nautical miles far from the previous confirmed northern limit (Gökova Bay) of the species. However, this report is totally questionable, as the photographs (Fig 3 in the paper by [90]) they provided cannot belong to the Sea of Marmara, but most probably to the Levantine or southern Aegean coast. The evidences from the photographs are; 1) The presence of the barren habitat in the photograph (absent in the Sea of Marmara): This is a habitat type specific to the Levantine Sea and the southern Aegean Sea mainly due to the intensive grazing of the herbivorous alien invasive species (e.g. *Siganus* spp.) on prevailing algal canopies [91]; 2) The presence of the thermophilic *Arbacia lixula* in the photograph (absent in the Sea of Marmara): the previous report of the species [50] was regarded as questionable [92]; 3) The presence of the thermophilic *Ostrea stentina* in the photograph (absent in the Sea of Marmara): The northern limit of this species is around Izmir Bay (central Aegean Sea) [93]; 4. The presence of the Red-Sea invader *Cerithium scabridum* in the photograph (absent in the Sea of Marmara): Gökçeada Island (north Aegean Sea) constituted its northern limit in the Aegean Sea [94]. The report of this species in Saros Bay [95] was also not considered in the present update with a similar reason.

In the 2011’s reports, the tropical Atlantic fish species that were classified as established (*Enchelycore anatina* and *Sphoeroides pachygaster*) and casual (*Carcharhinus altimus* and *Pisodonophis semicinctus*) alien species were excluded from the list as they were proved to be Atlanto-Mediterranean species that had entered the Mediterranean via the Gibraltar Strait without human assistance (see [8]). The boreal Atlantic pipefish *Syngnathus rostellatus* and dorab wolf-herring *Chirocentrus dorab* of the Indo-Pacific distribution that were treated as questionable in the 2011’s list was eliminated here as the former species is a misidentification of *S*. *tenuirostris* or *S*. *acus* (see [96]) and the latter species was identified solely based on larvae and eggs [8].

In the National Center for Biotechnology Information (NCBI) GenBank and in Barcode of Life Data System (BOLD), there are a total of 155 *Pterois miles* mitochondrial cytochrome c oxidase subunit 1 (COI) sequences obtained from Israel, Lebanon, Cyprus, Turkey and Greece [97–99]. The researchers investigated the *P*. *miles* populations in the Eastern Mediterranean and concluded that the invasion has occurred by multiple introductions. They have also included COI sequences of *P*. *miles*, *P*. *lunulata*, *P*. *russelii* and *P*. *volitans* from the Indian and Pacific Oceans to reveal the phylogenetic relationships. In none of the above-mentioned studies, occurrence of *P*. *volitans* has been validated in the Mediterranean Sea. Recently COI barcodes allegedly belonging to *P*. *miles* and *P*. *volitans* individuals collected from Iskenderun Bay was presented [100]. They have neither included any previously published genetic data in their study, nor have they deposited their sequences in NCBI or BOLD databases. The only accessible part of their genetic data is the sequence alignment shown in Table 2 of their publication [100]. They have given 9 COI haplotypes for *P*. *miles* and 7 haplotypes for *P*. *volitans*. However, the 81 basepair COI sequence which represents their *P*. *miles* specimens (Haplotype_1) does not match with “any” sequence in the GenBank when searched in NCBI Blast (Basic Local Alignment Search Tool) platform. Although COI sequences show interspecific divergence and used to discriminate species, it is a conserved gene, and any unrelated two fish species should show some homology in their COI sequences. On the other hand, the *P*. *volitans* COI sequences presented by [100] (Haplotypes 10–16) partially align, not with *P*. *volitans*, but various sequences of Bacteria, Yeast, Fungi, Plants, etc. in the GenBank, indicating a match by chance. Moreover, the interspecific genetic divergence value they presented (0.038178) cannot be correct, since the sequences they have shown for the two species are unrelated. In conclusion, it is very obvious that the COI sequences given by [100] are not related to the COI barcodes for any *Pterois* species. Also, it is hard to understand why authors have not compared their sequences with the previously published sequences found in the databases, or why they have not mentioned any of the genetic studies performed on the Mediterranean *Pterois* specimens. There is clear evidence that *P*.*miles* and *P*.*volitans* are two sibling species with extreme morphological resemblance, whose identification based primarily on simple meristic counts is often impossible [101, 102]. All previous records of *P*.*volitans* throughout the Mediterranean Sea [100, 103–105] are therefore refuted, all considered as misidentifications of *P*.*miles*.

The record of *Trachurus declivis* given from Mersin coasts (northern Levant) [106] is merely a misidentification of a xanthochromatic specimen of the native *T*. *trachurus*, both of which have quite distinct lateral line formations. In *T*.*declivis*, the lateral line bends downwards more suddenly; commences in a line with the 5th ray of 2nd dorsal and is entirely comprised within a space equal to that occupied by four finrays vs. the bend begins in a line with the commencement of the 2nd dorsal fin, and from its more gradual obliquity, extends over a space equal to that occupied by nine finrays in *T*.*trachurus* [107]. Moreover, dorsal accessory lateral line terminates below 5th to 11th (usually 7th to 9th) soft dorsal-fin ray in *T*.*declivis* [108] vs. 23rd to 31th soft dorsal fin ray in *T*.*trachurus* [109].

The Indo-Pacific soldier bream *Argyrops filamentosus* (Valenciennes, 1830) recorded from the Mediterranean Sea [110] is also a misidentified species. Associated with no photographs of a captured specimen, the morphometric characters presented by the authors provide enough data for revealing the error. The proportions of head length, predorsal length, prepelvic length, caudal peduncle depth and preorbital length in standard length of *A*.*filamentosus* [111] are by no means a match to the Mediterranean specimen. Although the preserved material was not examined herein for a precise identification, the morphometric characters most likely indicate the native *Pagrus caeruleostictus*.

An unvalid record of *Upeneus tragula* was given from Antalya Bay [112], based on a night-dive underwater photograph of a juvenile individual, but the photograph clearly belongs to a native *Mullus cf*. *surmuletus*. *Priacanthus hamrur* collected of Hatay (northeastern Levant) was misidentified as *P*. *sagittarius* [113], a species previously recorded from Turkey. Based on the recent review on Mediterranean Champsodontidae [114], previous records of *Champsodon capensis* and *C*.*vorax* [115, 116] are now considered as misidentifications of *C*.*nudivittis*. *Ruvettus pretiosus*, a circumtropical fish native to the Mediterranean Sea, was incorrectly treated as an alien species [117], naturally excluded herein.

Mitigation programmes of biological invasions require reliable national lists purified from errors [4], even so, inattentive taxonomic approaches are still present. A recent checklist of alien fish inventory of Turkey [118] includes huge number of erroneous entries, in which even taxa endemic to the Mediterranean Sea were inexplicably treated as alien species. Inclusion of the following native species to the alien inventory is evidently a mistake and should be excluded: *Petromyzon marinus*, *Alopias superciliosus*, *Mustelus punctulatus*, *Carcharhinus altimus*, *C*. *brevipinna*, *C*. *limbatus*, *Centrophorus granulosus*, *C*. *uyato*, *Squatina aculeata*, *Dasyatis marmorata*, *Mobula japanica*, *Notacanthus bonaparte*, *Enchelycore anatina*, *Dysomma brevirostre*, *Apterichtus caecus*, *Pisodonophis semicinctus*, *Nettastoma melanurum*, *Facciolella oxyrhyncha*, *Lepidion lepidion*, *Apletodon incognitus*, *Syngnathus rostellatus*, *Cephalopholis taeniops*, *Pomadasys incisus*, *Seriola fasciata*, *Corcyrogobius liechtensteini*, *Sphyraena viridensis* and *Sphoeroides pachygaster*.

### Cryptogenic species

The cyanobacter *Trichodesmium erythraeum*, the sipunculans *Apionsoma misakianum* and *Aspidosiphon mexicanus*, the bryozoan *Bugula neritina* and the amphipod crustacean *Monocorophium sextonae* that were classified as established alien species in the 2011’s species list, were moved to the cryptogenic category due to debate on their origins and alien status [4, 119–121].

In addition to the cryptogenic species listed in 2011 [7], we assigned here three more ascidian (*Botryllus schlosseri*, *Clavelina lepadiformis* and *Molgula manhattensis*) and one bryozoan (*Bugulina fulva*) species to the cryptogenic status, based on the recent detailed assessment [4]. The distributions of these species as well as their habitat and depth preferences were previously presented [122, 123].

### Impacts of invasive alien species

Developing mitigation measures against invasive species depends on a good understanding of their ecological, economic or health related influences, which has so far been documented only for a relatively low number of species. All alien species introduced to an ecosystem doubtless modifies the marine food webs to varying degrees, but available data denotes multifaceted impacts of invasive taxa transforming the decision-making process by governments or managers into a hard to solve situation. A recent comprehensive pan-European review proposed 87 marine taxa with high impact on ecosystem services or biodiversity [124], in which both negative and positive impacts were reported for 63 species, while 17 had only negative and 7 only had positive impacts. The most recent compilation of invasive species impacts observed at the Turkish coastline included information for over 40 species [7] and we present herein additional data obtained during the last decade.

**Fig 15:**
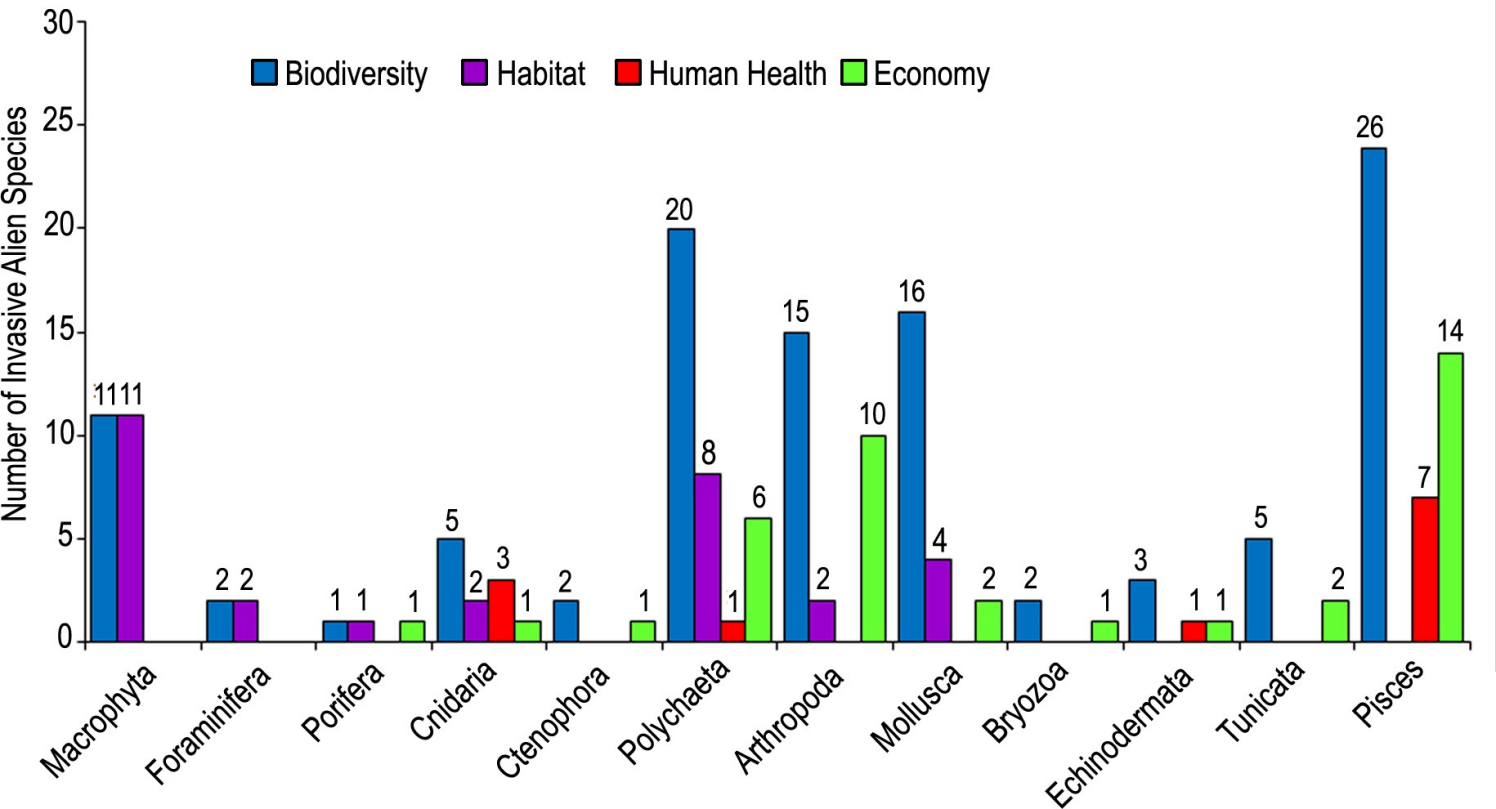
The number of invasive alien species affecting to native biodiversity, habitats, human health and economy (positive or negative).

A total of 105 invasive alien species have been reported from the coasts of Turkey, with documented and observed impacts on the major categories like biodiversity (all species), habitats (30 species), economy (both negative and positive contributions, 39 species) and human health (12 species). The impacts on these categories vary among taxonomic groups (Fig 15). Only Polychaeta (1 species, *Eurythoe complanata*), Cnidaria (3 species, *Rhopilema nomadica*, *Macrorhynchia philippina* and *Cassiopea andromeda*) and Pisces (7 species, *Siganus* spp., *Lagocephalus* spp., *Torquigener flavimaculosus* and *Pterois miles*) create problems in human health when touched (venomous chaetae, nematocysts or spines) or eaten (tetradotoxin in the flesh). Two groups (Cnidaria and Polychaeta) had impacts on all major categories.

#### Ecosystem and biodiversity impacts

**Fig 16:**
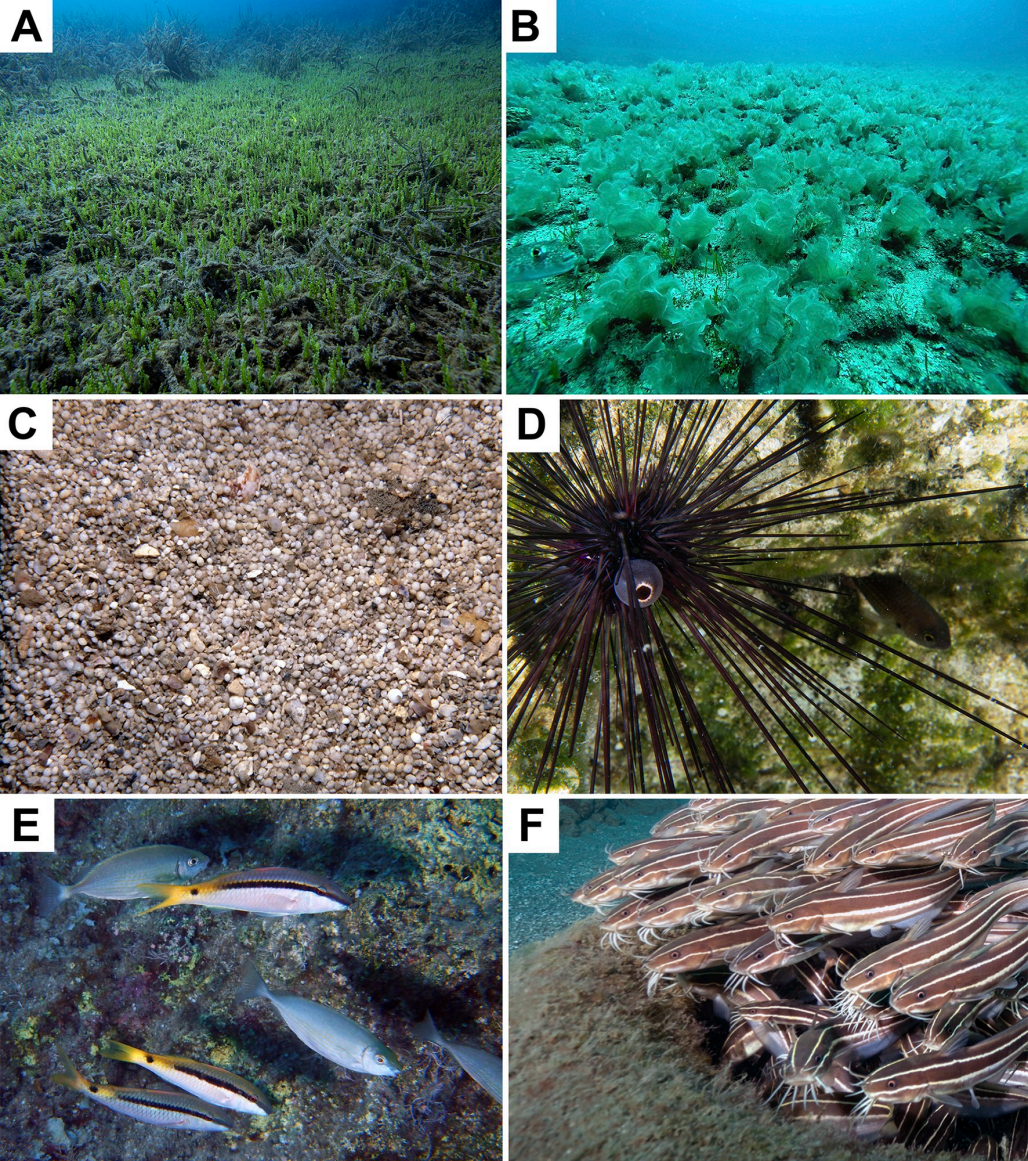
A—*Caulerpa cylindracea* observed at Ayvalık Islands Natural Park, forming dense populations at scars in the vicinity of *Posidonia oceanica* meadows (Photo: M.Bilecenoglu), B—*Stypopodium schimperi* forms dense populations at Turkish coasts (Photo: E. Taşkın), C—The famous “small pebble beach” at Kaş, Antalya Bay is currently covered entirely with *Amphistegina lobifera* tests (Photo: M.B.Yokeş), D–Several coastal fish species found shelter among long spines of *Diadema setosum* (Photo: M.Bilecenoglu), E—The Red Sea goatfish, *Parupeneus forsskali*, is currently the dominant alien mullid in several localities, often observed in association with *Siganus* spp. (Photo: M.Bilecenoglu), F–The venomous *Plotosus lineatus* is a marine health hazard (Photo: B.Selli).

There has been a considerable range expansion of *Caulerpa cylindracea* towards the northern Aegean Sea [125], with an increasing trend of its abundance. The maximum and mean algal coverage were estimated as 60% and 20%, respectively, between depths of 1 and 45 m at Ayvalık Islands Natural Park [126], but recent surveys proved a drastic raise of its coverage, extending to 100% in several localities (pers. obs. MB). The Ayvalık region is a touristic hotspot in Turkey, in which daily boat trips are a common recreational activity, but the serious damage caused by boat anchors leave scars behind, swiftly settled by *C*. *cylindracea* (Fig 16A).

Owing to the conspicuous role in the recipient ecosystems and taking the place of keystone species, the alien alga, *Stypopodium schimperi*, is considered as an invasive species in the eastern Mediterranean Sea [127]. Remarkable range expansion of the species towards the north Aegean Sea is evident in the last decade, which forms very dense populations along the shallow coastal strip (Fig 16B). The dead matte forms extended strips through the supralittoral zone, negatively influencing the existing faunal assemblages.

*Amphistegina lobifera* is the most abundant alien foraminifer species in the Mediterranean Sea, infamous for its ability to change the whole habitat type and coastal structure (Fig 16C). The species accumulates extensive sand and forms uncountable dense populations at the northern Levant, reaching to densities of up to 310000 ind.m^-2^ [7]. Current research indicated the immense impact of *A*. *lobifera* is continuing, whose tests form thick deposits of almost 60 cm in certain localities of Antalya Bay [128].

The Levantine population of *Penaeus kerathurus*, a native and commercially exploited species, has been severely hammered by intense fishing pressure and the invasion of its habitat by alien penaid species [129]. The penaeid shrimp catch composition in the region mainly consists of the alien species, such as *Penaeus pulchricaudatus*, *P*. *semisulcatus*, *P*. *hathor*, *Metapenaeopsis aegyptia*, *M*. *mogiensis consobrina*, *Trachysalambria palaestinensis*, *Metapenaeus monoceros* and *M*. *stebbingi* [130, 131], clearly indicating that *P*. *kerathurus* is being outcompeted.

The invasive longspine sea urchin *Diadema setosum* has recently been observed to provide refuge for coastal fish species (including the important keystone species, *Chromis chromis*, Fig 16D) against predators through their impenetrable long and venomous spines the northern Levant Sea [132]. This may be regarded as an ecologically positive impact, which should be closely monitored throughout its distribution range.

#### Socio-economic impacts

Among the invasive species, 39 species had direct impacts on the economy. Some species such as shrimps and edible fish (like *Nemipterus randalli*, *Siganus* spp., *Planiliza haematocheilus*, *Etrumeus golanii* and *Sphyraena* spp.) have positive contributions to fisher’s incomes, while a few such as *Rhopilema nomadica* and *Mnemiopsis leidyi* create a nuisance for commercial fisheries by clogging nets [7] or consume eggs or larvae of highly economic pelagic fish [133]. Eight invasive species have both positive and negative contributions to local economy. They are the species of high commercial value, which synchronously affect populations of other commercially important species. For example, the rapa whelk (*Rapana venosa*) is being intensively fished (positive impact), but have devastated beds of the native, highly economic mussel (*Mytilus galloprovincialis*) (negative impacts).

Several alien taxa have been commercially exploited in Turkey but obtained revenue can only be estimated for a few species. Capture fisheries of *R*. *venosa*, *C*. *sapidus*, *U*. *moluccensis* and *P*. *haematocheila* are regulated by the official fishery notifications (no. 5/1 and 5/2, valid until 31.08.2024), which set limitations on fishery methods, fishing seasons and minimum capture lengths. Started during the late 1980’s, there has been a growing up demand of the Rapa whelk from the Asian market and several processing plants were therefore established. According to official fishery statistics, almost 12,000 tons of *R*. *venosa* was produced on the coasts of the Sea of Marmara and Black Sea (Turkey) during 2019 and exported (as processed/frozen meat) to South Korea, Japan, China, Taiwan, USA and Vietnam, where over 18 million USD of income was obtained.

The Red Sea goatfish, *Parupeneus forsskali*, has an established population in the eastern Mediterranean Sea. First sightings off the northern Levant shores [15] were followed by other observations from Lebanon and Israel [134, 135]. An extreme population explosion was encountered in Turkey during the last few years, where the species become the dominant alien Mullid in certain localities (such as Antalya and Fethiye Bays, Fig 16E). The species has rapidly become a commercial catch for artisanal fishermen, sold with prices ranging 70 to 90 Turkish Lira (equivalent to 7–10 Euros). Competition with native *Mullus spp*. is quite likely, yet to be proved by further research.

#### Human health impacts

The long spine urchin, *Diadema setosum*, was first recorded in the Mediterranean Sea from Kaş, Turkey [136], which has rapidly invaded the northern Levant and southern Aegean Sea shores during the last decade. Unlike other native sea urchins, *D*. *setosum* can be even found over sandy substrates of very shallow depths (i.e., 0.1 m), making the species a potential threat to touristic activities. Several daily boat trips in Turkey only let the customers to swim once the long spine sea urchins from the area has been totally removed by the crew, especially at regions heavily infested by the species (for example Fethiye Bay). The slender venomous spines can inflict painful injuries, although a precise number of annual envenomation is not available.

All fin spines of *Pterois miles* are associated with venom glands, capable of producing intense pain. Given its enormous rate of invasion, the species possess high risk both to recreational and artisanal fishermen, as well as to tourists especially during the high season. No lionfish injuries have been reported from the Mediterranean Sea until 2018 [137], but there are at least two non-fatal injuries from Turkey appeared in local newspapers.

The established population of *Plotosus lineatus* is confined to Iskenderun Bay (Fig 16F), currently with no signs of westwards expansion. It is considered to be a dangerous fish, because of venom glands associated with the serrate spines on the dorsal and pectoral fins and skin secretions containing proteinaceous toxins [137], which is responsible from 10% of all venomous fish injuries reported by Israeli fishermen [138]. Its possible westwards range expansion is therefore a threat to human health.

Negative impacts of *Lagocephalus sceleratus* have quite well been documented in the Mediterranean Sea, yet unforeseen health related cases are still encountered. Following the first observations of biting cases from Antalya shores (i.e., a tourist was bit in the back, whilst a fisher suffered problems in one of his fingers; [139]), a traumatic amputation caused by a severe *L*. *sceleratus* bite in a child was observed, who eventually lost the distal part of her finger [140].

### Legislation

Environmental legislation in Turkey is mainly determined by the constitution, national laws, by-laws, regulations, notifications, international conventions and protocols. Protection of species and natural assets within their own ecosystems started legally in 1937 with the Forest Law and Terrestrial Hunting Law, but the Turkish Governments began focusing on environmental issues by the early 1980s. Since then, major steps have been taken towards harmonizing Turkey’s legislation both with EU acquis and with international standards, but still a huge effort is required to achieve the expected levels.

On national basis, Turkey does not currently have any laws specifically targeting marine alien or invasive species, but some indirect judgments are available mostly on biodiversity and environment issues, potentially linked to bioinvasions. The most prominent example is the Constitution of Turkey (adopted by the Parliament on 18.10.1982, law no. 2709), which mentions that the State shall ensure the protection of the historical, cultural and natural assets and wealth, and shall take supportive and promotive measures towards that end. The main legal framework is set out in Environment Law (no. 2872, dated 11.08.1983), emphasizing on the importance of protection of the environment, where all the activities threatening the biodiversity are prohibited.

General Directorate of Fisheries and Aquaculture (of Ministry of Agriculture and Forestry) is the main state organization responsible for the administration and regulation of fisheries and aquaculture. All activities are based on the Fisheries Law No. 1380 enacted in 1971, in which regulations and circulars are prepared to regulate fisheries. Article 25 of the Fisheries Law includes statements that may be linked to alien species (*all alive import and export of aquatic products are subject to permission the relevant Ministry*). For the first time in Turkey’s history, a recent incentive notification with short-term validity has been published in the official gazette (dated 02.12.2020, no: 31322), which permits artisanal fishermen to bounty hunt *Lagocephalussceleratus* for a price of 5 TL per fish caudal fin (0.52 Euros) during 02 to 31 December 2020. The notification supports hunting of one million *L*. *sceleratus* in total, but the targeted amount could not be reached and remained roughly at 46,000 individuals. The Ministry has unofficially announced that the same incentive approach will continue in 2021, by also including other alien pufferfish species.

There are two other laws defining border controls on plant and animal species entering/exiting Turkey (Agricultural Plant Protection and Agricultural Quarantine Law and Animal Health and Surveillance Law), where any kind of transfer is subjected to Ministry of Agriculture and Forestry regulations. Relevant laws concentrate mainly on terrestrial taxa and are loosely related to marine alien species, but they provide prominent baseline for import and export of species at the customs.

The Turkish National Biodiversity Strategy Action Plan was first published in 2008, in compliance with the Article 6 of CBD. Following the Decision 10/2 of Cop X/2, the NBSAP was reviewed and an updated version covering the 2018–2028 periods has been prepared [141]. Among the listed seven national objectives, the first one and its associated action directly focuses on alien and invasive species: *National Objective (1)—Pressures and threats on biodiversity and ecosystems will be determined*, *reduced to the lowest level or removed totally*; *Action 1*.*2*. *Studies on improving the measures for identifying*, *monitoring*, *and controlling the entrance routes of invasive species and alien species and preventing entrance and habitation thereof will increasingly be continued*.

Since invasive alien species are a global threat that clearly requires international cooperation and actions, there are many international agreements (binding/non-binding) and regulations referring directly or indirectly to alien species [142]. Those ratified by Turkey are as follows: Convention on Biological Diversity, Ramsar Convention (The Convention on Wetlands of International Importance especially as Waterfowl Habitat), CITES (The Convention on International Trade in Endangered Species of Wild Fauna and Flora), The International Convention for the Control and Management of Ships’ Ballast Water and Sediments (BWM Convention), Convention on the Conservation of European Wildlife and Natural Habitats (Bern Convention), and Protocol Concerning Specially Protected Areas and Biological Diversity in the Mediterranean (Barcelona Convention). According to the Turkish legislation, ratification of the above-mentioned conventions is not sufficient alone without the implementation regulations (yet to be prepared by the responsible Ministries) have come into force.

### Management

No successful story has yet been written in the effective management of marine invasive alien species in the Mediterranean, and the world’s seas as well. Although certain studies have been carried out for the killer alga *Caulerpa taxifolia* to cease its invasion (manual removal and application of a cloth soaked in copper salts) in the western Mediterranean (see [143]), all of them ended in disappointment. Despite of several international conventions ratified; Turkey has been progressing very slowly to manage on-going invasions by alien species. The recent fishery notification entered into force aims to create a pressure on the existing stocks of *L*. *sceleratus*, which is responsible from the huge negative impact on artisanal fisheries (by damaging fishing gears), as well as on food web dynamics (through predator-prey relationships) and human health. Since the bounty hunting is limited to 1 million of fish and only valid between 02 to 31 December 2020, the expected impact (if any) will only be of minor magnitude and evidently far from effective management of alien species. However, it would be also on the agenda to create a management plan later with the experiences to be gained from this initiative.

Among all other alternatives, the pre-border management (i.e., the management of vectors) seems to be the most effective way of alien species management [144]. Turkey signed and ratified (Law Number: 6531, date: 8.4.2014) the International Convention For the Control and Management of Ship’s Ballast Water and Sediments, 2004 (BWM). This is an important step towards preventing translocations of species by shipping activities. According to the D1 standard of the BWM Convention, vessels need to perform ballast water exchange (at least 95% capacity) at least 50 nautical miles from the nearest land and in waters at least 200 m deep. According to the D2 standard, vessels may only discharge ballast water that contains viable organism within specified limits (by treating ballast water) [145]. This might considerably decrease risks of species introductions via ballast water of ships, which was previously poured into sea near ports where tolerant introduced species can easily survive and establish [146, 147].

In the eastern Mediterranean, where Turkey is located, a little can be done for the management of invasive alien species as long as the Suez Canal is widely open. As stated in this study, the Red Sea species comprised more than half (57%) of the number of established and casual species reported from Turkey and this ratio varies considerably among seas. In accordance with the expansion of the Suez Canal, which was fully completed in 2016 [6]; sea-level change through the canal over years [148]; decrease in the salinity level of hyperhaline lakes (Bitter Lake) through the canal, and the reduction of the fresh-water outflows of the Nil River after the construction of the Aswan Dam, which acted as a freshwater barrier at the gateway (Port-Said) to the Mediterranean [149]; and global climate changes [150] a significant increase in the number of introduced species will likely to occur, yet to be meticulously and closely monitored. Therefore, alien species management in Turkey, in a sense, is associated very closely with the management of the Suez Canal itself. It is not possible to protect the Mediterranean habitats from the Red Sea originated species invasions without deploying smart engineering solutions on the canal such as saltwater-freshwater barriers. The importance of international cooperation in the management of Lessepsian species is increasing than ever before, as their increasing profound impacts and incalculable costs on the biodiversity and socio-economy cannot be overcome by any individual country. Regardless under which umbrella it would be (e.g., Barcelona Convention, UN Biodiversity Convention), the Mediterranean countries should sit around a table to start a discussion on the effective management of the Suez Canal and to create solution plans with alternatives. In this context, the time has come to pass a proposal to compensate the ecosystem services lost by the countries due to the invasion of Red Sea species from the country operating the Suez Canal.

## Conclusion–The way forward

The coasts of Turkey, which accounted for almost 65% of the total number of alien species reported from the Mediterranean Sea till now, will be undoubtedly subjected to new alien species entrance from now on. For this reason, in defiance of critically evaluated available information on the taxonomy and distribution characteristics of alien species, producing an up-to-date alien species inventory on the coasts within a certain time period (preferably once in every decade) will allow us a better understanding of the diversity, trends and establishment success of alien species. Surrounded by four seas of different oceanographic features, it is essential to prepare and implement different management plan for each sea of Turkey, taking cognizance of the vectors and invasion strategies of the introduced species. As a result of investigations by academic institutions scattered along the coastal cities of Turkey, a mass of information has been produced so far on the alien species distribution pattern in certain areas such as İskenderun Bay and İzmir Bay. In addition, the relatively long-term, on-going monitoring projects such as the Integrated Pollution Monitoring Studies covering all seas of Turkey financed by the Ministry of Environment and Urbanization in the last decade have enabled keeping the information up to date. However, in order to monitor and surveil alien species at least in hot-spot areas, implementation of long-term specific projects in an eastern Mediterranean country like Turkey, which lose habitats and native biodiversity hence causing a few billion US dollar financial loss annually, is a prerequisite rather than arbitrariness. In addition, apart from those on the identification and distribution of alien species, future multi-oriented studies towards the invasion bio-ecology of alien species and their impacts on the prevailing ecosystems (especially in the food chain) are needed for allowing us a better understanding of irreversible changes these species have created in the Mediterranean ecosystems.
